# Atad2 is a generalist facilitator of chromatin dynamics in embryonic stem cells

**DOI:** 10.1093/jmcb/mjv060

**Published:** 2016-08-19

**Authors:** Yuichi Morozumi, Fayçal Boussouar, Minjia Tan, Apirat Chaikuad, Mahya Jamshidikia, Gozde Colak, Huang He, Litong Nie, Carlo Petosa, Maud de Dieuleveult, Sandrine Curtet, Anne-Laure Vitte, Clothilde Rabatel, Alexandra Debernardi, François-Loïc Cosset, Els Verhoeyen, Anouk Emadali, Norbert Schweifer, Davide Gianni, Marta Gut, Philippe Guardiola, Sophie Rousseaux, Matthieu Gérard, Stefan Knapp, Yingming Zhao, Saadi Khochbin

**Affiliations:** 1 INSERM, U823; Université Grenoble Alpes; Institut Albert Bonniot Grenoble, F-38700 Grenoble, France; 2 The Chemical Proteomics Center and State Key Laboratory of Drug Research, Shanghai Institute of Materia Medica, Chinese Academy of Sciences, Shanghai 201203, China; 3 Nuffield Department of Clinical Medicine, University of Oxford, Structural Genomics Consortium, Old Road Campus Research Building, Roosevelt Drive, Oxford OX3 7DQ, UK; 4 Nuffield Department of Clinical Medicine, University of Oxford, Target Discovery Institute (TDI), NDM Research Building, Roosevelt Drive, Oxford OX3 7FZ, UK; 5 Ben May Department of Cancer Research, The University of Chicago, Chicago, IL 60637, USA; 6 Université Grenoble Alpes/CNRS/CEA, Institut de Biologie Structurale, 38027 Grenoble, France; 7 Institute for Integrative Biology of the Cell (I2BC), CEA, CNRS, Univ. Paris-Sud, Université Paris-Saclay, CEN Saclay, 91191 Gif-sur-Yvette, France; 8 CIRI, International Center for Infectiology Research, EVIR team, INSERM U1111, CNRS, UMR5308, Université de Lyon-1, ENS de Lyon, Lyon, France; 9 INSERM, U1065, Centre Méditerranéen de Médecine Moléculaire (C3M), équipe ‘contrôle métabolique des morts cellulaires’, Nice 06204, France; 10 Boehringer-Ingelheim RCV GmbH & Co KG, Dr. Boehringer Gasse 5-11, A-1121 Vienna, Austria; 11 CNAG–Centre for Genomic Regulation (CRG), Baldiri Reixac 4, 08028 Barcelona; Universitat Pompeu Fabra (UPF), Barcelona, Spain; 12 INSERM, U892; Centre de Recherche sur le Cancer Nantes Angers and UMR_S 892; Université d'Angers; Plateforme SNP, Transcriptome & Epigénomique; Centre Hospitalier Universitaire d'Angers, Angers 49000, France

**Keywords:** FACT, epidrug, germ cells, cancer drug target, histone turnover, Pax3, histone chaperone

## Abstract

Although the conserved AAA ATPase and bromodomain factor, ATAD2, has been described as a transcriptional co-activator upregulated in many cancers, its function remains poorly understood. Here, using a combination of ChIP-seq, ChIP-proteomics, and RNA-seq experiments in embryonic stem cells where *Atad2* is normally highly expressed, we found that Atad2 is an abundant nucleosome-bound protein present on active genes, associated with chromatin remodelling, DNA replication, and DNA repair factors. A structural analysis of its bromodomain and subsequent investigations demonstrate that histone acetylation guides ATAD2 to chromatin, resulting in an overall increase of chromatin accessibility and histone dynamics, which is required for the proper activity of the highly expressed gene fraction of the genome. While in exponentially growing cells Atad2 appears dispensable for cell growth, in differentiating ES cells Atad2 becomes critical in sustaining specific gene expression programmes, controlling proliferation and differentiation. Altogether, this work defines Atad2 as a facilitator of general chromatin-templated activities such as transcription.

## Introduction

The advent of small molecule inhibitors targeting bromodomains has made it possible to tackle signalling processes that follow chromatin acetylation, especially in pathological settings, such as cancer. Although the proof of concept has been clearly established in the case of BET bromodomains (Picaud et al., 2013; Filippakopoulos and Knapp, 2014; Sanchez et al., 2014; Schaefer, 2014), the utility of targeting bromodomains in most other proteins awaits additional investigation (Philpott et al., 2014). ATAD2 is a remarkably conserved protein present in lower and higher eukaryotes (Cattaneo et al., 2014). In many eukaryotes, two closely related genes encode *ATAD2* paralogs (Cattaneo et al., 2014). In human, they are designated as *ATAD2A* and *ATAD2B*/*KIA1240*. All *ATAD2* orthologs share a characteristic N-terminal AAA ATPase domain and a C-terminal bromodomain. The almost systematic upregulation of *ATAD2A* in many unrelated solid human tumours (Caron et al., 2010) and its association with poor prognosis in various cancers including lung cancer (Caron et al., 2010), breast cancer (Caron et al., 2010; Kalashnikova et al., 2010), hepatocellular carcinoma (Wu et al., 2014; Yang et al., 2014), and ovarian carcinoma (Wan et al., 2014) strongly suggest that *ATAD2A* overexpression favours malignant transformation and cancer progression.

Additionally, several molecular studies have identified ATAD2A as a transcriptional co-regulator acting on cancer/proliferation-promoting factors such as oestrogen and androgen receptors (Zou et al., 2007, 2009), E2F transcription factors (Revenko et al., 2010) and Myc (Ciro et al., 2009; Boussouar et al., 2013). Taken altogether, these data suggest that ATAD2A could be a relevant drug target for bromodomain inhibitors, and early chemical starting points targeting the bromodomain have been identified (Chaikuad et al., 2014). Despite these studies, the function of ATAD2 in a ‘normal’ physiological setting has never been addressed. To conform with most of the literature, we refer to ATAD2A as ATAD2 throughout this text.

In order to investigate the function of ATAD2 in its physiological context, we used a bioinformatics-based strategy to identify the origin of normal ATAD2 expression. This approach shows that *ATAD2* is not only highly expressed in male germ cells, as we reported previously (Caron et al., 2010), but also normally predominantly active in embryonic stem (ES) cells, prompting us to undertake a comprehensive study of Atad2 function in this latter setting. To this end, we first used a knock-in approach to introduce three C-terminal tags to the endogenously expressed Atad2 and then combined ChIP-seq, ChIP-proteomics, and RNA-seq approaches to generate comprehensive sets of data on Atad2 function. Additional functional studies allowed us to characterize the normal function of Atad2, and to show that it is a general auxiliary factor targeting acetylated histones and facilitating chromatin-templated processes by maintaining chromatin accessible. Our findings also suggest that this function is particularly critical in sustaining differentiation-specific gene expression and cell growth.

## Results

### ATAD2 is predominantly expressed in embryonic stem cells

**Figure 1:**
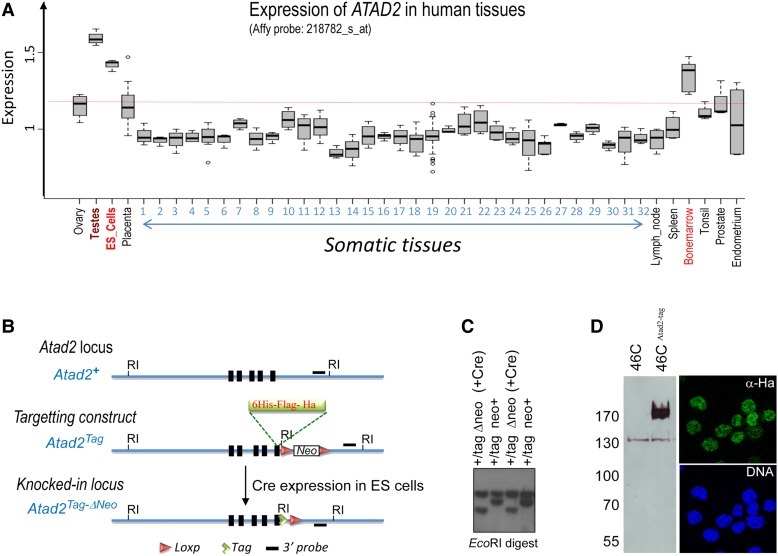
ATAD2 is predominantly expressed in male germ and ES cells: Knock-in strategy to introduce TAP tags at the Atad2 C-terminal region. (**A**) Raw .CEL files were downloaded from the GEO website (http://www.ncbi.nlm.nih.gov/geo/) corresponding to data from 351 samples of normal human tissues, including a large series of adult somatic and germline tissues (334 samples from GSE3526), placenta (14 samples from GSE18809, GSE7434, and GSE9984), and embryonic stem cells (3 samples from GSE9440). The data were normalized together using the RMA algorithm for summarization and quantile normalization, and the expression of the *Atad2* gene in each of the indicated tissues is shown as box plots. The numbers from 1 to 32 indicate the following tissues, respectively: adipose tissue, adipose omental, adipose subcutaneous, adrenal gland cortex, amygdala, bronchus, cervix, colon caecum, coronary artery, oesophagus, heart atrium, heart ventricle, kidney cortex, kidney medulla, liver, lung, mammary gland, myometrium, neuro, nipple cross section, oral mucosa, pharyngeal mucosa, pituitary gland, salivary gland, saphenous vein, skeletal muscle, stomach cardia, stomach fundus, stomach pyloric, thyroid gland, tongue main corpus and trachea. (**B**) The map of the 3′ end of *Atad2* gene encompassing the last coding exons is shown. The indicated DNA segment containing the TAP tag 6xHis-Flag-Ha, upstream of a *LoxP* site-flanked-*Neo* cassette in frame with *Atad2* last exon, was introduced in the ES cell genome by homologous recombination. The expression of Cre recombinase in ES cells led to the deletion of the *Neo* cassette. The *Eco*RI sites and the 3′ probes used for Southern analysis (black lines) are indicated. (**C**) A Southern blot revealed by the probe indicated in **B** shows ES cell *Atad2* region before and after expression of Cre recombinase (+Cre) and excision of the *Neo* cassette (Δneo). (**D**) Protein extracts prepared from ES cells expressing Atad2-tag (46C^Atad2-tag^) or not (46C) were probed by an anti-Ha (left panel), and cells were stained with this antibody to detect Atad2-tag by immunofluorescence (right panel).

Our previous investigation of *ATAD2/Atad2* gene expression pattern and protein accumulation showed that the gene is normally highly expressed in male germ cells and that it is also frequently abnormally active in many cancers, similar to many other testis-specific genes (Caron et al., 2010). In order to explore the normal pattern of *ATAD2* expression in more details, we carried out a recently described bioinformatics approach (Rousseaux et al., 2013), which enabled us to estimate ATAD2 expression in large series of Affymetrix transcriptomic data from various normal and non-tumoral human tissues. This analysis revealed that *ATAD2* is predominantly expressed in male germ cells and, to a lesser extent, in ES cells, as well as in some haematopoietic tissues (bone marrow), whereas its expression level is low or null in most normal adult somatic solid tissues (Figure 1A). Hence, *Atad2* belongs to a group of genes predominantly expressed in germ cell/stem cell (Wang et al., 2015). Therefore, in order to investigate Atad2 function in its normal expression setting, we used mouse embryonic stem (ES) cells and combined the power of next-generation sequencing and proteomics approaches. To maximize the reliability of these ‘omics' approaches, we set up a tandem purification protocol enabling a drastic reduction of the background noise and high confidence identification of Atad2-associated genomic regions and proteins.

The recombineering approach (Liu et al., 2003) was used to introduce three tags, Flag, Ha, and His, at the C-terminal part of the endogenous Atad2 protein (Figure 1B). Clones showing successful homologous recombination (Figure 1C) were established, and one of them was chosen for further studies. Initial analyses showed that the protein was expressed and could easily be detected by an anti-Ha antibody in a total cell lysate, as well as *in situ*, where the protein was exclusively nuclear (Figure 1D).

### Genome-wide mapping of Atad2 chromatin interactions in ES cells

Studies in cancer had shown that ATAD2 is a transcription co-activator acting together with a variety of transcription factors such as E2F, oestrogen and androgen receptors, and Myc (Boussouar et al., 2013). We mapped all genomic sites associated with Atad2 in ES cells to see whether Atad2 could also be associated with specific transcriptional regulatory elements and/or with a particular gene expression programme in these cells.

For this purpose, we used the immunoprecipitation following tandem affinity purification (TAP) protocol, which proved to purify Atad2 very effectively with almost no background (see below, the ChIP-proteomics approach). Cross-linked sheared chromatin fragments underwent two rounds of ChIP. After the first ChIP using an anti-Flag antibody, Atad2-bound fragments were released with an excess of Flag peptides and then used for the second round of ChIP using an anti-Ha antibody. Atad2-associated genomic DNA fragments were then sequenced. The parental 46C ES cells, expressing the non-tagged form of Atad2, were used in parallel as a control and did not show any significant peak enrichment under the same ChIP conditions.

**Figure 2:**
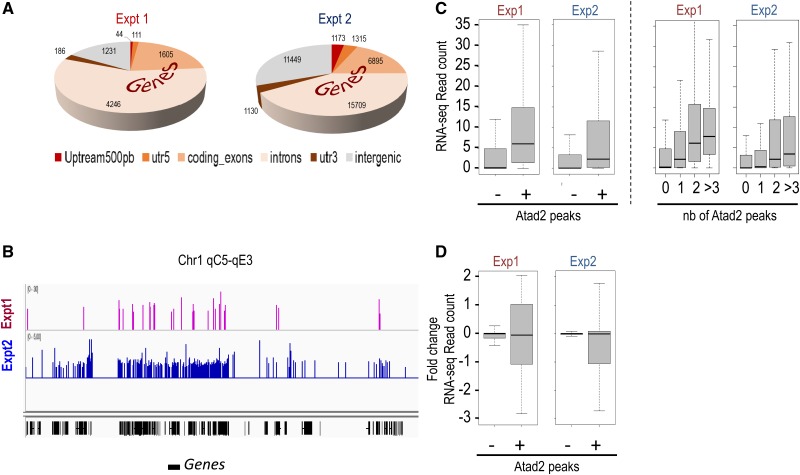
Visualization of Atad2–genome interaction by ChIP-seq. (**A**) In two different sets of experiments, the chromatin of 46C or 46C^Atad2-tag^ ES cells was subject to immunoprecipitations. In the first experiment (Exp1), sheared formaldehyde-crosslinked chromatin followed by a tandem purification protocol was used, whereas in the second experiment (Exp2), DNA sequencing was performed after an anti-Ha immunoprecipitation of non-crosslinked MNase-digested chromatin fragments (see Figure 3A). In both cases, the genomic annotations of the sequenced fragments are presented as a pie chart. Peak numbers corresponding to these annotations are indicated. (**B**) An example illustrating the enrichment in Atad2 peaks of gene-rich regions of the genome is shown for the two ChIP-seq experiments. (**C**) The expression levels of two groups of genes, those associated with Atad2 peaks (+) and those not associated with Atad2 peaks (−), are shown as box plots for both ChIP experiments (left panel: Exp1 and Exp2, respectively). The expression levels increase significantly with the number of Atad2 peaks (right panel: ANOVA *P*-values <0.001). The gene expression levels were determined from RNA-seq data from wild-type 46C^Atad2-tag^ cells, treated with a scrambled siRNA (see below, **D**). (**D**) 46C^Atad2-tag^ cells were treated with two active anti-*Atad2* siRNAs and an inactive siRNA. Each RNA sample was sequenced twice, and the efficiency of Atad2 knockdown was verified by comparing Atad2 mRNA read counts from control cells and cells treated with either anti-*Atad2* siRNA. The indicated fold changes correspond to the ratios of read counts of knockdown vs. wild-type cells, all using the corresponding read counts. The fold change ratios of gene expressions after Atad2 knockdown compared with control ES cells in both ChIP experiments are shown for the two groups of genes: those associated with Atad2 peaks (+) and those not associated with Atad2 peaks (−).

The analysis of Atad2-associated genomic peaks revealed a clear bias towards gene-containing regions compared with intergenic regions (Figure 2A, Exp1), but no particular enrichment was observed in transcription start sites (TSS), promoters, or enhancers. Indeed, >80% of the Atad2 peaks were found over gene-containing regions (including coding exons (26%) and intronic regions (55%)), and only a minor fraction was found within 5 kb upstream from TSS (2%). Figure 2B shows an example of Atad2 peaks associated with gene clusters and their relative depletion in intergenic regions (Exp1).

Since chromatin crosslinking affects antigen recognition, and the TAP procedure may lead to important losses in the final DNA recovery, we also performed a native single anti-Ha ChIP and analysed the Atad2-associated regions (Exp2). Indeed, we reasoned that, due to a very tight association of Atad2 with chromatin (see below, the ChIP-proteomics approach), crosslinking should not be necessary to reliably isolate Atad2-associated chromatin.

In all these experiments, the parental 46C cell line was used as a control. The second experiment corroborated the results presented above. A clear enrichment of Atad2 peaks in gene-containing regions of the genome was observed (65%), mainly encompassing coding exons and intronic regions (Figure 2A, Exp2). Both experiments show that, although enriched over the gene-containing regions of the genome, the peaks do not show any specific localization within these regions (Figure 2A and B, Exp2).

We then considered the characteristics of Atad2-associated genes. First, using a RNA-seq approach, we established ES gene expression levels and then compared the global expression of Atad2-associated genes with genes devoid of Atad2 peaks. Strikingly, both ChIP experiments demonstrated that genes associated with Atad2 present a much higher transcriptional activity compared with those devoid of Atad2 (Figure 2C, left panel). Moreover, the levels of transcriptional activity, measured by the number of RNA-seq reads in ES cells, increased significantly in genes with higher Atad2 peak density (Figure 2C, right panel).

Next, we used a siRNA approach to knock down Atad2 and measure the resulting variations in gene expression by RNA-seq. Interestingly, the knockdown of Atad2 using two different siRNAs specifically affected the transcription of Atad2-associated genes, but not the expression of genes devoid of Atad2, suggesting that Atad2 intervenes in sustaining the transcriptional activity of the associated genes (Figure 2D).

We also analysed the generated RNA-seq data to see whether any of the known mouse genome repetitive sequences showed a differential expression with Atad2 knockdown. Data compiled in Supplementary Figure S1 show that none of the considered repeat elements presented a deregulated expression.

Overall, these data demonstrate that, although Atad2 is neither a transcription factor nor a specific transcriptional co-regulator, it associates with transcriptionally active genomic domains to contribute to the maintenance of gene activity.

### ChIP-proteomics analysis of Atad2-bound chromatin regions

The ChIP-seq data suggested that Atat2 could be a ‘generalist’ factor, rather than a specific ‘transcription factor’, associated with active chromatin regions such as genes showing high transcriptional activities. To support this hypothesis, we purified Atad2-bound nucleosomes using the tandem affinity protocol and identified the associated factors.

**Figure 3:**
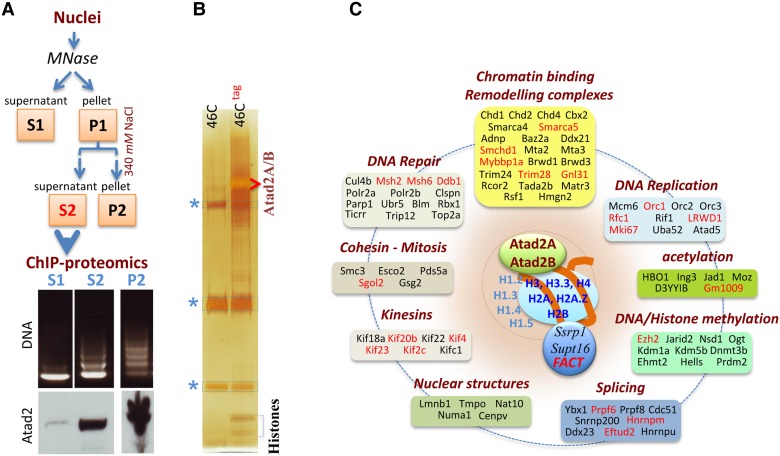
Characterization of Atad2–chromatin interaction by ChIP-proteomics. (**A**) The chart flow indicates the important steps in nuclear extract preparation. The ethidium bromide-stained gel shows the state of chromatin digestion by MNase (DNA panel), and the presence of Atad2 in the corresponding fractions is revealed by western blot (WB panel). (**B**) A tandem affinity purification (TAP) protocol was used to purify Atad2-tag-bound nucleosomes (fraction S2). The same approach was used on the same amount of extracts from the parental 46C ES cell line. The immunoprecipitated materials were visualized on a silver-stained gel (see also Figure 4B). Asterisks indicate Ig bands corresponding to the second immunoprecipitation. All gel segments containing bands present in 46C^tag^ ES cell sample were cut out from both 46C^tag^ and 46C lanes, respectively (see Supplementary Figure S2 for a better visualization of the analysed gel segments), and analysed by MS. (**C**) Atad2-nucleosome associated factors were grouped into the indicated functional categories (see Supplementary Table S1A). An independent tandem affinity purification of Atad2 was performed, where, after Flag peptide elution of immunoprecipitated materials, an immobilized Ha antibody cross-linked to Dynabeads was used for the second immunoprecipitation, and the immunoprecipitated materials was eluted using a SDS-PAGE loading buffer (see Figure 4B as an example of the set of proteins obtained following this protocol). The eluted material was loaded on a SDS-PAGE gel and underwent short migration and isolated in a single gel segment, which was then submitted to MS analysis. As above, a strictly same procedure was carried out on C46 cells, and the samples were analysed in parallel. Proteins from both C46^tag^ and C46 were identified in red and reported in Supplementary Table S2. The results of Atad2 ChIP-proteomics shown here were also compared with Yta7 ChIP-proteomics data (Lambert et al., 2010) organized in the same way (Supplementary Figure S3).

Pilot experiments were performed and revealed that a tight association of Atad2 with chromatin hampered the preparation of soluble nuclear extracts containing Atad2. This problem was overcome by extensive micrococcal nuclease (MNase) digestion. Figure 3A shows that nucleosomes released after MNase treatment were devoid of Atad2 (S1 fraction), while a significant amount of Atad2 was released along with mononucleosome-enriched chromatin fragments after treatment of nuclei with a buffer containing 340 mM NaCl (S2 fraction). The rest of Atad2 remained in the insoluble nuclear material (P2 fraction).

**Figure 4:**
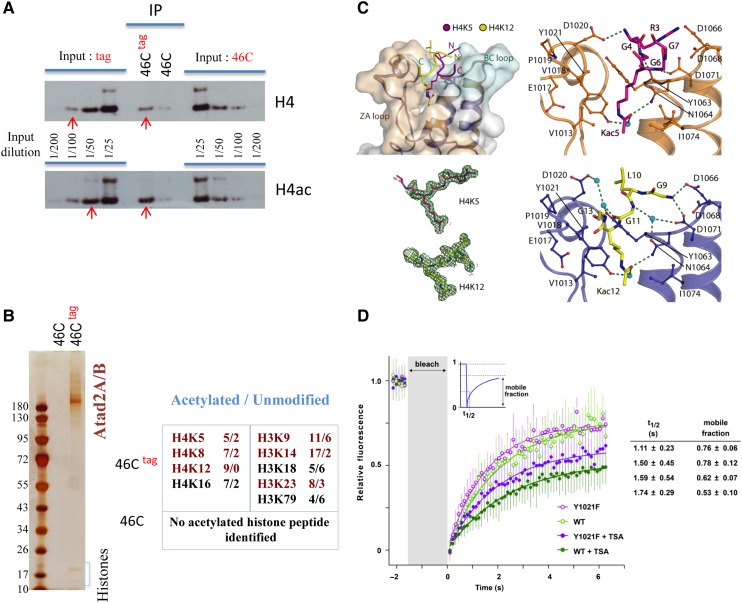
Atad2 is targeted to hyperacetylated nucleosomes. (**A**) Protein extracts from Atad2-tagged (46C^tag^) and parental (46C) ES cells were immunoprecipitated with the anti-Flag antibody, and the precipitated materials were analysed by western blot probed by anti-H4 and anti-acetylated H4 (indicated). The western blot also contained the indicated dilutions of the input materials used for IP. The relative intensity of H4 and H4ac bands in 46C^tag^ IP compared with the corresponding signal in the input was determined. Bands with similar intensities are indicated with red arrows. (**B**) Histones identified after TAP tag purification of Atad2 (indicated) were further analysed for detection of acetylation by MS (note that here a cross-linked anti-Ha was used, therefore no Ig bands are visible). In the indicated 46C^tag^ and 46C samples, the count of peptides with or without acetylation (acetylation/unmodified) at specific sites is given. No significant acetylation was detected in the histones from the parental 46C cells. (**C**) The respective structures of the ATAD2 bromodomain bound to either H4K5 or H4K12 acetylated peptide were determined. The upper left panel displays the surface representation of the ATAD2 bromodomain. The two co-crystallized H4 peptides are superimposed and highlighted by different colours as indicated. The main structural elements are highlighted. The lower left panel indicates the 2Fo-Fc electron density map contoured at 2 σ around the peptide ligands. The upper right panel shows details of the interaction of H4K5ac peptide with the ATAD2 bromodomain, and the lower right panel details the interaction of H4K12ac peptide with this domain (hydrogen bonds are shown as dotted lines). (**D**) Wild-type GFP-ATAD2 or GFP-ATAD2 bearing the Y1021F mutation in its bromodomain was expressed in Cos7 cells, treated or not with TSA. After photobleaching, the recovery of GFP fluorescence was monitored. Ten datasets for each experiment were individually fitted. The eight giving the best fitting statistics were used to calculate the half-life (t_1/2_) of fluorescence recovery and mobile fractions (right table). The mean and standard deviation of the time points from the eight datasets are plotted on the left.

The tandem purification protocol for Atad2 recovery was then applied on the S2 fraction. The first immunoprecipitation was carried out using anti-Flag M2 beads followed by elution with Flag peptide. The eluted material was then immunoprecipitated using an anti-Ha antibody. The corresponding silver-stained gel showed efficient recovery of Atad2 and associated histones as well as diverse proteins of various molecular weights (Figures 3B and 4B and Supplementary Figure S2). All the gel segments containing bands visible on the silver-stained gel were then cut from both samples for analysis by mass spectrometry (MS) (Supplementary Figure S2).

The results confirmed that the most intensely stained bands correspond to Atad2. Interestingly, both Atad2A and Atad2B were detected in these bands with a similar relative abundance (Supplementary Table S1A). The other intensely stained bands correspond to the core histones, confirming that Atad2 is indeed tightly bound to nucleosomes.

In perfect agreement with the ChIP-seq data showing an association of Atad2 with active gene transcription, a large number of Atad2-associated factors are members of chromatin remodelling complexes or mediate histone/DNA modification or RNA splicing (Figure 3C). In addition to transcription, we also found factors involved in DNA replication, DNA repair, and mitosis (Figure 3C, Supplementary Table S1A), indicating that Atad2 is generally present at all sites where various factors act on chromatin.

In order to validate these findings, we performed an independent tandem purification of Atad2-tag and MS-based identification of associated proteins. This time, we performed a second immunoprecipitation using an immobilized anti-Ha antibody (cross-linked to Dynabeads), and the immunoprecipitated material was recovered with a SDS-PAGE loading buffer, shortly run on a gel, and used as a whole for MS analysis in a single gel segment.

The second approach, although less comprehensive than the first one, confirmed the presence of important chromatin-acting factors that were also identified in the first ChIP-proteomics experiment (indicated in red in Figure 3C and listed in Supplementary Table S2).

The identified factors were largely involved in various chromatin-templated functions, i.e. DNA replication, DNA repair, mitosis, as well as in transcription, chromatin remodelling, and splicing (Figure 3C). Interestingly, in two independent studies, which aimed at establishing the proteomics of replicating DNA, ATAD2 was found in the list of proteins enriched on replicating chromatin (Sirbu et al., 2013; Alabert et al., 2014), further supporting the relevance of our identified Atad2-associated factors and its association with sites of dynamic chromatin alterations.

Atad2 is a conserved factor, sharing striking sequence and domain homology with its budding yeast ortholog, Yta7 (Cattaneo et al., 2014). Since yeast factors were also identified after Yta7 ChIP-proteomics, we wondered whether these Yta7-associated factors would fall within the same functional categories as the Atad2-interacting factors identified here. The data from Yta7 ChIP-proteomics published by Lambert et al. (2010) were recovered, classified into functional categories, and compared with our Atad2 ChIP-proteomics data (Supplementary Figure S3).

This comparison reveals striking functional similarities between Atad2 and Yta7. Both factors are tightly bound to nucleosomes, and associated with the FACT histone chaperone complex, as well as the same functional categories of actors mediating chromatin remodelling, DNA replication, repair, and transcription.

Overall, the data from these two ChIP-proteomics experiments are in excellent agreement with the data not only from our two ChIP-seq approaches (Figure 2), but also from Yta7 ChIP-proteomics (Supplementary Figure S3), highlighting the specific binding of Atad2 at sites of active chromatin where diverse factors are in action.

### Atad2 interacts with acetylated chromatin regions

To determine whether chromatin binding by Atad2 is related to histone acetylation, we compared the level of histone H4 acetylation in histones that co-purified with Atad2 with the general H4 acetylation level in the input chromatin. Figure 4A shows the presence of only background signal when the anti-Flag IP was performed in the parental cells, whereas, as expected, strong signals for both H4 and acetylated H4 were detected in Atad2-tag immunoprecipitated materials. A comparison among various amounts of input showed that the immunoprecipitated H4 corresponded to 1/100 of the total input, whereas the H4ac IP corresponded to ∼1/50 of the H4ac in the input. This indicates that the chromatin associated with Atad2 shows at least a 2-fold enrichment in acetylated H4.

In order to assess the acetylation levels of Atad2-bound chromatin more precisely, the acetylation of histones recovered after the tandem purification of Atad2 was also analysed by MS. This analysis revealed an enrichment of core histones acetylated at various sites over non-modified histones (Figure 4B, see Supplementary Figure S4 and Table S1B and C for details).

To elucidate the structural basis of the ATAD2–histone interaction, we co-crystallized the ATAD2 bromodomain with peptides derived from the acetylated H4 tail. We previously found the preference of ATAD2 bromodomain for multiple acetylated histone tails (Filippakopoulos et al., 2012), and we had observed that mouse Atad2 preferentially binds to H4K5ac (Caron et al., 2010). Consequently, we focussed on H4 acetylated on K5 and K12. Hence, the purified ATAD2 bromodomain was crystallized with H4 tail peptides monoacetylated at either of these two sites.

Both structures revealed the canonical acetyl-lysine binding mode, characterized by a hydrogen bond between the acetyl-lysine carbonyl and the conserved asparagine N1064, as well as a water-mediated hydrogen bond to Y1021 (Figure 4C). For the two acetylated H4 peptides, residues R3 to G7 and G9 to G13 are well resolved in the electron density maps (Figure 4C, lower left). As described for other bromodomains, water molecules occupy the bottom of the acetyl-lysine binding site (Filippakopoulos et al., 2012). However, apart from these conserved interactions, which are also shared with the binding mode of the isolated acetyl-lysine residue (Chaikuad et al., 2014), the binding mode of the H4K5ac- and H4K12ac-containing peptides outside the central binding pocket is highly diverse. The H4K12ac-containing peptide forms only backbone or water-mediated interactions with the ATAD2 bromodomain, and the hydrophobic residue L10 adopts a seemingly unfavourable position next to a polar surface pocket (Figure 4C, lower right). In contrast, the interactions with H4K5ac were more optimal, containing a number of main-chain and side-chain hydrogen bonds, as well as a salt bridge interaction between H4R3 and D1066 (Figure 4C, upper right).

This structural analysis highlights a critical role for Y1021 within the Atad2 bromodomain for acetylated histone binding. In order to verify the role of the ATAD2 bromodomain in chromatin targeting, we introduced an inactivating mutation (Y1021F) in the bromodomain. By using a fluorescence recovery after photobleaching (FRAP) approach, we monitored the dynamics of the wild-type protein and the bromodomain mutant before and after the induction of histone hyperacetylation by TSA treatment. Prior to these experiments, we confirmed that the Y1021F mutation indeed prevents ATAD2 bromodomain from binding to acetylated histones (Supplementary Figure S5).

We also compared publically available ChIP-seq data mapping the occurrence of H3K9 and H3K27 acetylation in mouse ES cells with our Atad2 ChIP-seq data. Within Atad2-bound regions, we compared the amount of Atad2 binding between acetylated and non-acetylated regions. Supplementary Figure S6 shows that, in agreement with our proteomic and FRAP data, Atad2 is preferentially associated with regions enriched for H3 acetylation.

In agreement with the role of the bromodomain in targeting acetylated chromatin regions, TSA-induced histone hyperacetylation resulted in a significant reduction of ATAD2 mobility (Figure 4D), presumably due to the immobilization of ATAD2 on acetylated chromatin. Consequently, ATAD2 harbouring an inactive bromodomain showed a significantly higher mobility compared with wild-type ATAD2. Interestingly, the mobility observed in the absence of TSA was not completely blocked by the bromodomain mutation (Figure 4D). This partial effect might be explained by the ability of ATAD2 to multimerize with endogenous ATAD2 bearing a functional bromodomain.

### Atad2 sustains a globally open and dynamic chromatin in ES cells

Combined ChIP-seq and ChIP-proteomics identified Atad2 as a generalist factor associated with active chromatin regions where a variety of regulatory factors mediate gene transcription and other energy demanding chromatin alterations. Most interestingly, the ChIP-proteomics approach also identified two subunits of FACT (Figure 3C), a known histone chaperone involved in various chromatin-templated processes (Formosa, 2013). Altogether, our observations suggested that Atad2 could also help the function of many factors acting on chromatin. Accordingly, one way to sustain this ‘auxiliary’ function would be to permanently maintain chromatin accessible to other factors.

**Figure 5:**
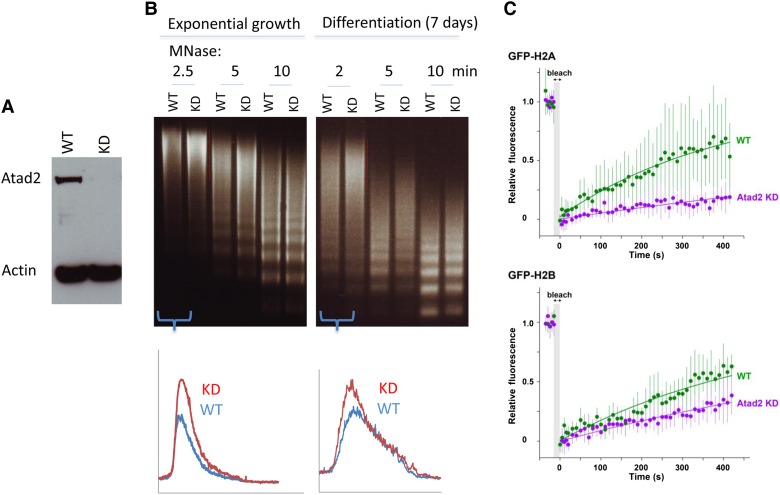
Atad2 activity underlies ES cell overall chromatin accessibility and dynamic histone exchange. (**A**) 46C^tag^ cells were infected with viral particles expressing an active anti-*Atad2* shRNA and an inactive shRNA for the establishment of stable *Atad2* knockdown cells. (**B**) Nuclei from control (WT) and stable Atad2 knockdown (KD) ES cells in exponential growth or after 7 days of culture in the absence of LIF were prepared and digested for the indicated times with MNase. The lower panel shows the comparative densitometric scans of the first MNase digestion times. (**C**) WT and KD ES cells were transfected with GFP-H2A and GFP-H2B expression vectors, as indicated, and histone turnover was measured by FRAP. FRAP data for each histone and cell type are presented as indicated. Plotted data represent the mean and standard deviations of eight experiments. Knockdown of Atad2 results in ∼4-fold and 2-fold increase in the apparent half-life of FRAP for GFP-H2A and GFP-H2B, respectively, reflecting the lower mobility of these proteins upon Atad2 depletion.

To test this hypothesis, we first generated a stable knockdown of Atad2 using ES cells infected with an anti-Atad2 short hairpin (sh) RNA expressing vector carrying viruses. Another pool of infected cells expressing an inactive anti-Atad2 shRNA was used as a control (Figure 5A). The general accessibility of ES cell chromatin to MNase was then tested in both exponentially growing ES cells and cells after 7 days of LIF withdrawal-induced differentiation. Figure 5B shows that, as predicted, Atad2 knockdown significantly increased the resistance of chromatin to MNase digestion in both non-differentiated and differentiated ES cells. To test whether a dynamic exchange of histones was favoured by maintaining chromatin accessible, GFP-tagged H2A and H2B were individually expressed in ES cells containing wild-type or reduced levels of Atad2, and histone turnover was monitored by FRAP as a readout for chromatin dynamics. As expected and in perfect agreement with accessibility measurements, Atad2 knockdown resulted in a decreased turnover of both H2A and H2B (Figure 5C). These experiments fully support the conclusions drawn from ChIP-seq and ChIP-proteomics data and show that Atad2 is acting primarily on active regions of the genome to maintain an accessible and open chromatin.

### Atad2 activity sustains cell proliferation during ES cell differentiation

**Figure 6:**
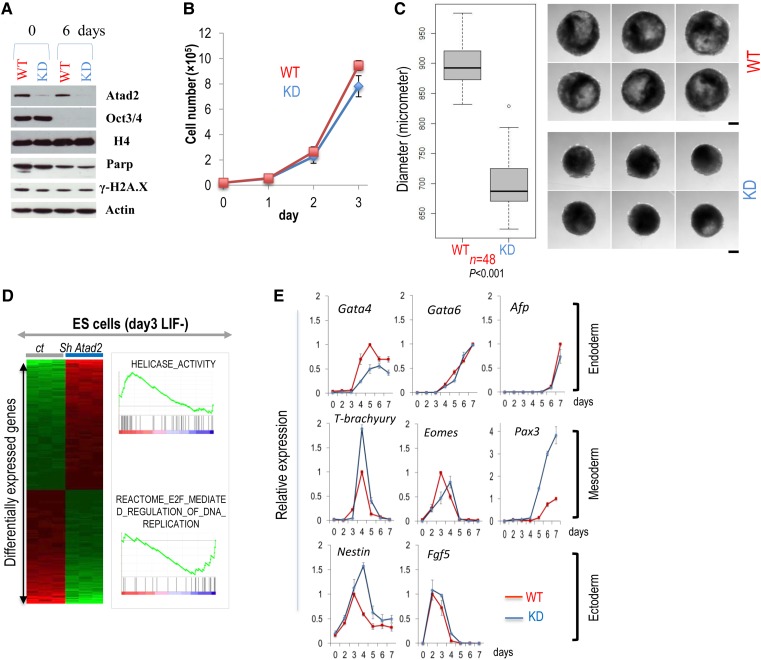
Atad2 is involved in ES cell differentiation. (**A**) Both control ES cells (WT, expressing an inactive shRNA) and cells with reduced amounts of Atad2 (KD, stably expressing an active anti-*Atad2* shRNA) were seeded in LIF-depleted media for 6 days. Extracts were prepared and the indicated proteins were visualized. (**B**) WT and KD ES cells were seeded, and cell growth was monitored as a function of time. (**C**) WT and KD ES cells were cultured in the differentiation medium, and the diameter of EBs was measured after 6 days of differentiation and presented as box plots. Forty-eight EBs were measured for each condition, and the *P*-value (Mann–Whitney) corresponding to size differences between the two cell populations is indicated. The corresponding values are presented in Supplementary Table S3. Six representative EBs for each condition are shown. (**D**) Control ES cells and cells with reduced amounts of Atad2 were placed in the differentiation medium for 3 days. RNA was prepared and the transcriptome was revealed following hybridization on Illumina chip. The heatmaps show genes differentially expressed between control cells (ct) and cells with Atad2 knockdown (Sh Atad2). Illustrations of GSEA categories of genes whose expression is regulated by Atad2 knockdown are shown. (**E**) WT and KD ES cells were placed in LIF-depleted media for 7 days. Cells were harvested every day and RNA were prepared. The expression of the indicated genes, specific representatives of differentiation into the three germ layers, was monitored by RT-qPCR and normalized with respect to the expression of *actin*. The data shown are representative of two independent experiments. Standard deviations are shown.

Despite alterations in chromatin dynamics and gene expression (Figures 2D and 5), ES cells with a stable Atad2 downregulation (Figures 5A and 6A) could be kept in culture and their growth was not significantly affected (Figure 6B). However, after removal of LIF and the subsequent downregulation of Oct4 on Day 6 of differentiation (Figure 6A), the cells lacking Atad2 showed defective growth ability, as demonstrated by the reduced size of the embryoid bodies (EBs) (Figure 6C). The absence of PARP cleavage or H2A.X phosphorylation excluded the possibility of increased cell apoptosis during differentiation in the absence of Atad2 (Figure 6A).

These experiments indicate that, in contrast to ES cells undergoing exponential growth, Atad2 is required to maintain cell proliferation in the absence of LIF. This observation is reminiscent of the behaviour of *ATAD2*-expressing cancer cells, which are not dependent on ATAD2 during their exponential growth phase but need ATAD2 to proliferate in the absence of serum (Ciro et al., 2009).

In order to investigate whether the above observations could be attributed to an Atad2-dependent alteration of gene expression in differentiating ES cells, a transcriptomic analysis was performed after 3 days of differentiation, prior to the downregulation of Oct4 and subsequent effects on gene expression (Figure 6D). A gene set enrichment analysis (GSEA) showed that, in the absence of Atad2, genes of the helicase functional category were upregulated, suggesting a possible compensation for the absence of Atad2, while a gene category involved in stimulating cell proliferation regulated by the E2F transcription factor was downregulated after Atad2 knockdown, in agreement with the loss of the ability of cells to proliferate properly (Figure 6D).

This finding is in line with previously reported cooperation of Atad2 with E2F in cancer cells (Boussouar et al., 2013), and suggests that cells during differentiation exhibit an increased requirement for Atad2 activities compared with exponentially growing cells.

Accordingly, we also performed RT-qPCR to analyse the expression levels of several specific genes, which are considered as markers for differentiation toward the three germ layers, during 7 days after LIF removal in wild-type and *Atad2*-knockdown cells. Figure 6E demonstrates that the absence of Atad2 affects gene expression along the three differentiation pathways, showing either a modified expression timing or an enhanced or reduced expression, i.e. *Gata4* (endoderm), *T-brachyury*, *Eomes*, *Pax3* (mesoderm), and *Nestin* (ectoderm).

In conclusion, these analyses support the hypothesis of a generalist action of Atad2 in enhancing chromatin-templated functions. In exponentially growing ES cells with a particularly hyperdynamic chromatin, the contribution of Atad2 may appear relatively subtle, whereas in differentiating ES cells where the proliferation capacity of cells decreases, the action of Atad2 on chromatin becomes critical in sustaining specific functions such as the establishment of particular gene expression programmes.

## Discussion

Here we provide a clear view of the functions of Atad2 in its physiological context of expression. Atad2 is highly expressed in ES and germ cells where chromatin is highly dynamic and undergoes dramatic remodelling (Meshorer et al., 2006; Melcer et al., 2012; Boskovic et al., 2014). Combined ChIP-seq and ChIP-proteomics approaches and subsequent investigations unambiguously identify Atad2 as a generalist enhancer of chromatin dynamics, recruited at acetylated chromatin regions, such as actively transcribed genes, where chromatin-templated processes are in action. A detailed inspection of Yta7 ChIP-proteomics data revealed striking functional similarities between factors associated with both Atad2 and Yta7, among which the histone chaperone complex FACT was found associated with Atad2 and Yta7.

Interestingly, all the findings reported here on Atad2 recall a series of independent publications highlighting various properties and functions of the histone chaperone FACT. Indeed, FACT is conserved and associated with DNA replication, DNA repair, and transcription (Formosa, 2013). FACT expression is restricted to stem cells: it is not expressed in normal adult mammalian cells, but was found overexpressed in a variety of aggressive cancers (Garcia et al., 2011, 2013; Gasparian et al., 2011). Additionally, the genome-wide mapping of Atad2 interaction with chromatin shown here revealed a profile that is very similar to that reported previously for FACT (Garcia et al., 2013). Finally, FACT was found recruited to sites of DNA repair where it accelerates the exchange of histones (Dinant et al., 2013). The common property of FACT and Atad2, as well as Yta7, is their ability to interact with histones. However, in contrast to FACT, Atad2 bears a bromodomain capable of specifically guiding the protein toward acetylated histones where it could act as a molecular motor thanks to its AAA ATPase domain. These latter characteristics indicate that Atad2 belongs to a particular generalist functional category different from known histone chaperones that could also include another AAA ATPase domain factor, p97/VCP. Indeed, p97 is involved in a myriad of cellular functions (Yamanaka et al., 2012), including genome stability (Vaz et al., 2013), and is nicknamed the ‘Swiss army knife’ of cell biology (Baek et al., 2013). It uses the energy obtained from its AAA ATPase activity in a large number and unrelated cellular activities. However, the important difference with p97 is again that Atad2 possesses a bromodomain, which guides the protein toward a more specialized and chromatin-specific activity.

Early studies of ATAD2 in cancers, considering a series of cancer-related transcription factors, such as Myc, E2F, or androgen and oestrogen receptors, led previous investigators to define ATAD2 as a transcriptional co-activator (Boussouar et al., 2013). The comprehensive investigations of Atad2 properties reported here suggest that these observations actually reflect one facet of an otherwise general ‘facilitator’ ATAD2, which contributes to providing a highly accessible and dynamic chromatin to these factors. Indeed, it is possible that in cancer cells, the oncogenic transcription factors use ATAD2 to facilitate their action on chromatin and the resulting malignant transformation would impose a privileged direct or indirect interaction between these factors and ATAD2.

An intriguing fact is the dispensable nature of ATAD2 in cancer cells (Ciro et al., 2009) as well as in exponentially growing ES cells reported here. Considering the remarkably conserved nature of Atad2 (Cattaneo et al., 2014) and its associated chromatin factors, one could expect that Atad2 is essential to some specific chromatin-templated events such as replication. Indeed, our ChIP-proteomics approach revealed the presence of a large number of critical DNA-replication factors associated with nucleosome-bound Atad2. Additionally, two independent groups that established the proteomics of replicating DNA reported the enrichment of ATAD2 on genomic zones undergoing replications (Sirbu et al., 2013; Alabert et al., 2014). However, despite strong evidence for a functional implication of Atad2 in DNA replication, in neither cancer cells (Ciro et al., 2009) nor exponentially growing ES cells, the absence of Atad2 affected cell growth.

These observations, along with the fact that ATAD2 has never been identified in the numerous large-scale screenings for essential cellular functions, strongly support our conclusion that Atad2 essentially ensures a global ‘helping’ function, which in growing cells can be compensated for by the combined action of other more specialized factors directly acting on chromatin. However, in differentiating ES cells, when chromatin dynamics dramatically decreases after Oct4 downregulation (Meshorer et al., 2006; Melcer et al., 2012), we demonstrate that Atad2 becomes important for sustaining cell growth. One can assume that, due to a general downregulation of cell proliferation factors under these conditions, the requirement for Atad2 increases and the helper action of Atad2 becomes more important to ensure the ongoing chromatin activities. These observations also help us understanding why *ATAD2* is almost systematically activated in a variety of cancers (Caron et al., 2010). Indeed, its helper activity could promote the action of various factors, including oncogenic ones, to drive malignant transformation.

In summary, this comprehensive functional study of Atad2 in ES cells allows us to designate this factor as a new generalist factor capable of ‘preparing’ chromatin for an adequate response to a wide range of factors.

## Materials and methods

### Knock-in of a TAP tag in Atad2 in ES cells

The gap-repair recombineering technique (Liu et al., 2003) was used with long homology arms for constructing the targeting vector for homologous recombination in ES cells. Briefly, the retrieval vector was generated by mixing PCR product 1 (left arm, *Not*I/*Spe*I), PCR product 2 (right arm *Spe*I/*Bam*HI), and the plasmid PL253 (*Not*I/*Bam*HI). To generate the *Neo*-targeting vector, the PL452 vector was modified by inserting the TAP tag (6His-Flag-Ha). An *Asc*I site was inserted in frame to allow the cloning of 5′ homology arms as *Sal*I/*Asc*I fragments in the PL452^tag^ vector. Electro competent SW102 cells containing *Atad2* BAC, which had been induced for recombination by prior growth at 42°C for 15 min, were electroporated with the *Spe*I-linearized retrieval vector. A 10-kb *Atad2* fragment spanning the last exon was sub-cloned. Then the TAP tag was introduced along with a *Neo* cassette into the sub-cloned DNA to create the knock-in targeting vector.

For gene targeting, 20 µg of *Not*I-linearized TAP tag targeting vector (PL253) DNA was electroporated (250 V with a capacitance of 500 µF) into 20 × 10^6^ 129SV 46C ES cells, which were growing on mitomycin-C-inactivated MEFs. Colonies were selected in ESC medium containing 15% fetal bovine serum and LIF in DMEM with G418. Hundreds of neomycin-resistant clones were picked and analysed by Southern blot with a 3′ external probe.

### Antibodies

The following antibodies were used in this study: Anti-Ha.11 (MMS-101R, Covance) for immunoblotting, anti-Ha (Clone 3F10, Roche) for immunoprecipitation, anti-Flag (F3165, Sigma-Aldrich), anti-Oct3/4 (s5279x, Santa-Cruz), anti-β-actin (A5441, Sigma-Aldrich), anti-histone H4 (ab31827, Abcam), anti-acetyl histone H4 (06-866, Millipore), anti-pADPr (reveals PARP, 1020, Tulip BioLabs), and anti γ-H2A.X (2212-1, Epitomics).

### Plasmid constructs

pCMV6-h*ATAD2*-GFP (RG218291, Cliniscience) was used as a template to PCR-amplify full-length *ATAD2*-ORF and the mutant *ATAD2*-ORF-Y1021F fragments, which were cloned in the expression vector pEGFP-C1 (Clontech Laboratories, Inc.) for FRAP analysis.

### ESCs culture, differentiation, and FRAP experiments

46C ESCs (a gift from Philippe Avner) were cultured in DMEM medium containing LIF (ESG1107, Millipore), non-essential aminoacids, antibiotics (Penicillin and streptomycin), β-mercaptoethanol, and supplemented with 15% fetal bovine serum (Invitrogen), on 0.1% gelatin-coated tissue culture dishes at 37°C in CO_2_ incubator. For differentiation, LIF was withdrawn from ESCs medium, and cells were allowed to form spheroids (EBs). For cell cycle analysis of EBs, 1 × 10^6^ ES cells were seeded in bacterial Petri dishes and cultured for different times. For measuring the size of EBs, 1000 ES cells were seeded in 96-well round bottom low cell binding plate (Thermo Scientific) to produce homogeneous EBs and cultured for 6 days. At the end of the experiment, EBs were harvested for analysis, and their diameter was calculated using ImageJ software.

FRAP experiments were performed after expression of GFP-tagged histones in control or siAtad2 KD ESCs, as previously described (Montellier et al., 2013).

### Generation of stable Atad2 knockdown ESC

Non-targeting or *Atad2* targeting oligonucleotides were cloned into the lentiviral vector pLKO.1-puromycin (Addgene) for shRNA expression and packaged as described (Levy et al., 2010). 46C-Atad2-tagged ESCs were transduced with lentiviral particles for 24 h and further cultured for an additional 5 days in the presence of puromycin (2 µg/ml) for selection. Stable pooled cells were analysed for Atad2 depletion by western blot using Ha antibody or by RT-qPCR. The sequences of the oligonucleotides are given below.

### ATAD2 expression pattern in normal tissues and RNA purification, reverse-transcription, and real-time quantitative PCR analysis

Affymetrix transcriptomic data from a variety of data sets available at the GEO site were used to establish the origin of *ATAD2* expression. Samples from available published data of normal human tissues (GSE3526, GSE11350, GSE15431, GSE18809, GSE6872, GSE7434, and GSE9994) were used to identify and characterize *ATAD2* expression levels. The Affymetrix expression data files (.CEL) corresponding to the respective studies were downloaded from the GEO website (http://www.ncbi.nlm.nih.gov/geo/). The data were normalized using the RMA algorithm for summarization and quantile normalization.

ESCs were harvested with TRI Reagent (Sigma), and total RNA was isolated following the manufacturer's instructions. RNA was reverse-transcribed with the SuperScript™ III First-Strand Synthesis SuperMix (Invitrogen). Real-time quantitative PCR analysis of cDNA was performed on MxPro 3005P (Stratagene) with the Brilliant SYBR Green QPCR Master Mix (Agilent Technologies). Primers for cDNA analysis are listed below.

The sense-strand sequences in Table 1 were used to clone shRNA oligonucleotides in pLKO.1 vectors for stable infection of ESCs; Atad2 shRNA GGTTGTAGCTCCTCCAAAT has already been tested and validated (Caron et al., 2010) and AAACCTCGTCACCAGAGA is an inactive shRNA sequence. For all transient transfection experiments for Atad2 silencing, the following primers were designed using Eurogentec software: siRNA#1 Atad2 CGCCCAGCAATTATATTCT, siRNA#2 Atad2 GCGGATATTAAGTCAATCT, and siScrambled.

**Table 1:** Primers used in quantitative RT-PCR experiments.

	Forward	Reverse
*Atad2*	TCCAACTGGAGAATTTGTATGC	ACTGCTTGCTCCATTTTCTGA
*Oct4*	TCTTCTGCTTCAGCAGCTTG	GTTGGAGAAGGTGGAACCAA
*Actin*	CTAAGGCCAACCGTGAAAAG	ACCAGAGGCATACAGGGACA
*U6*	CTCGCTTCGGCAGCACA	AACGCTTCACGAATTTGCGT
*Gata6*	GACGGCACCGGTCATTACC	ACAGTTGGCACAGGACAGTCC
*Gata4*	GTCGTAATGCCGAGGGTGA	TCCTTCCGCATTGCAAGAG
*AFP*	TGAAGCAAGCCCTGTGAACTC	TCAGAAAACTGGTGATGCATAGC
*T-Brachyury*	CAGCCCACCTACTGGCTCTA	GAGCCTGGGGTGATGGTA
*Pax3*	TCCATCCGACCTGGTGCCAT	TTCTCCACGTCAGGCGTTG
*Eomes*	CCGGGACAACTACGATTCCA	ACCTCCAGGGACAATCTGATG
*Nestin*	CTGCAGGCCACTGAAAAGTT	GACCCTGCTTCTCCTGCTC
*Fgf5*	ACCCGGATGGCAAAGTCAA	CAATCCCCTGAGACACAGCAA

### Transcriptomic analysis after sh Atad2 and Gene Sets Enrichment Analysis (GSEA)

Transcriptomic analyses, comparing ES cells stably expressing anti-*Atad2* shRNA versus cells expressing control shRNA, in LIF (+) ES cells (six replicates for each condition) and Day 3 LIF (−) differentiating ES cells (three replicates for each condition), were performed using the Illumina MouseWG-6 v2.0 expression beadchip technology and interpreted as described (Gaucher et al., 2012). A GSEA was performed using the gene symbols of the differentially expressed genes with the online tool provided by the Broad Institute (http://www.broadinstitute.org/gsea/index.jsp), as described in Le Bescont et al. (2015).

### Tandem affinity purification of Atad2

A total of 20 confluent Petri dishes (15 cm^2^) for 46C and 46C^tag^ cell lines were used for Atad2 purification shown in Figure 3. Fresh ESC pellets (4 ml each) were resuspended in hypotonic buffer (10 mM Hepes-NaOH (pH 7.9), 1.5 mM MgCl_2_, 10 mM KCl, 1 mM DTT, 340 mM sucrose, and 1× Complete protease inhibitor (Roche)) and then disrupted by the addition of 10% NP-40 (final 0.1%). After incubation on ice for 5 min, nuclei were isolated by centrifugation at 500× *g* for 10 min. Nuclei were then resuspended in MNase buffer (20 mM Hepes-NaOH (pH 7.9), 100 mM KCl, 10% glycerol, 2 mM CaCl_2_, 1 mM DTT, and 1× Complete protease inhibitor) and chromatin was digested by micrococcal nuclease S7 (MNase; Roche) at 37°C for 10 min. After centrifugation at 500× *g* for 10 min, the pellets were resuspended in IP buffer (20 mM Hepes-NaOH (pH 7.9), 340 mM NaCl, 10% glycerol, 0.2 mM EDTA, 0.1% NP-40, 1 mM DTT, and 1× Complete protease inhibitor) and incubated with rotation at 4°C for 1 h. The supernatants were recovered as a starting material for immunoprecipitation by centrifugation at 21000× *g* for 10 min, followed by filtration through a 0.45-µm membrane filter. The protein extracts were incubated with M2 Flag beads (Sigma-Aldrich) at 4°C for 4 h. The beads were then washed five times with IP buffer and the proteins were eluted twice with 0.3 µg/µl of 3× FLAG peptide (Sigma-Aldrich) by incubation at 4°C for 30 min.

The elution fractions were used for the second immunoprecipitation. At this point, two protocols were followed using free or crosslinked anti-Ha antibodies. Eluates were incubated with 0.16 µg of free anti-Ha or Dynabeads cross-linked anti-Ha at 4°C for 1 h. Dynabeads protein G (Life Technologies) were then added and further incubated at 4°C for 1 h (for free anti-Ha). After washing the beads three times with IP buffer, the proteins bound to the beads were recovered by Laemmli sample buffer. The proteins were separated by SDS-PAGE for MS. In the case of second protocol, anti-Ha antibody was cross-linked to Dynabeads protein G with BS^3^ [Bis (sulfosuccinimidyl) suberate] according to the manufacturer's instructions, and after precipitation, washed and proceeded as above.

### Proteomic-based approaches

#### Nano-HPLC/MS analysis of Atad2 associated proteins

Tryptic peptides from each band were dissolved in HPLC buffer A (0.1% formic acid, 2% acetonitrile, 98% water). Samples were then injected into a manually packed reversed phase C18 column (170 mm×79 µm, 3-µm particle size, Dikma, China) coupled to Easy nLC 1000 (Thermo Fisher Scientific). Peptides were eluted from 5% to 80% buffer B (0.1% formic acid, 90% acetonitrile, 10% H_2_O) in buffer A with 30 min gradient at a flow rate of 300 nl/min. The fractions were analysed by using an Orbitrap Elite mass spectrometer in a top 20 data-dependent mode. For full MS spectra, the scan range was *m*/*z* 350–1300 with a resolution of 240000 at *m*/*z* 400; for MS/MS scan, ions with charge state 1–3 in each full MS spectrum were sequentially fragmented by collision-induced dissociation with normalized collision energy of 35%. The dynamic exclusion duration was set to be 20 sec, and the isolation window was 2 *m*/*z*.

#### Analysis of MS raw data

All MS raw files were analysed by Mascot software (version 2.4.0) against the database Uniprot Mouse. Acetylation (protein N-term) and oxidation (M) were specified as variable modifications. Mass error for parent ion mass was ±10 ppm, and fragment ion was ±0.5 Da. Enzyme was specified as trypsin with three maximum missing cleavages. Peptide ion score cut-off was 20, and protein score cut-off was 30. When there were only one or two matching peptides for a protein, spectrum was manually checked (Chen et al., 2005).

For histone modification analysis, the MS raw files of histone bands were analysed by Mascot software (version 2.4.0). Acetylation (K), methylation (K), dimethylation (K), and trimethylation (K) were specified as variable modifications. Mass error for parent ion mass was ±10 ppm with fragment ion as ±0.5 Da, and enzyme was trypsin with five maximum missing cleavages. Peptide ion score cut-off was 20, and acetylated peptides were checked manually. The ratio of acetylated/unmodificated peptides was based on spectrum counting.

### ChIP-seq and RNA-seq

Two different protocols were used to perform ChIP-seq, one after formaldehyde crosslinking and a tandem purification of Atad2-associated regions and the other following a native ChIP protocol with a single anti-Ha immunoprecipitation.

In the case of native ChIP, the protocol for processing immunoprecipitated DNA fragments and sequencing were the same as previously published (Gaucher et al., 2012).

For formaldehyde-crosslinking ChIP, 5 × 10^6^ Atad2-tagged ESCs were fixed for 10 min at room temperature with 1% formaldehyde added to the tissue culture medium. Cells were then washed three times with PBS, and collected in solution I (15 mM Tris–HCl (pH 7.5), 0.3 M sucrose, 60 mM KCl, 15 mM NaCl, 5 mM MgCl_2_, 0.1 mM EGTA). An equal volume of solution II (same as buffer I with 0.6% Igepal) was then added to the cells, mixed, and incubated on ice for 10 min. Cells were then centrifuged at 1000 rpm for 5 min and collected in MNase buffer (20 mM Tris–HCl (pH 7.5), 0.34 M sucrose, 15 mM KCl, 60 mM NaCl, 1 mM CaCl_2_). Chromatin was next fragmented by micrococcal nuclease (MNase) digestion: 90 units of MNase (New England Biolabs) were added per 10 million cells. After 10 min incubation at 37°C, MNase digestion was stopped by incubating the cells on ice and adding 4 mM EDTA (final concentration) and protease inhibitors (complete, Roche). Cells were then subjected to four cycles of sonication (20 sec on; 40 sec off) using a Diagenode bioruptor and centrifugated at 10000× *g* for 10 min at 4°C. The supernatant, which contains the fragmented chromatin was collected (total volume ∼2 ml), and incubated overnight at 4°C with 80 µl of anti-Flag M2-Agarose beads (Sigma-Aldrich) in TEGN buffer (20 mM Tris–HCl (pH 7.5), 150 mM NaCl, 3 mM MgCl_2_, 0.1 mM EDTA, 10% glycerol, 0.01% Igepal). The beads were washed eight times with 10 ml TEGN, and resuspended in 300 µl of TEGN buffer. A hundred microliters of Flag peptide (4 μg/μl) were added to the beads, which were rotated at room temperature for 5 h to allow the release of bound chromatin by peptide competition. The supernatant was collected and incubated overnight at 4°C with 40 μl of anti-Ha agarose beads (Sigma-Aldrich, monoclonal antibody HA-7). The beads were then washed eight times with 10 ml TEGN, and elution was performed by competition with a Ha peptide as described above. The eluted chromatin was adjusted to 0.2 M NaCl, heated at 65°C overnight to reverse the formaldehyde-mediated crosslink, digested with proteinase K, and extracted by phenol-chloroform. The DNA was precipitated with ethanol in the presence of glycogen, and solubilized in water. The purified DNA was size-selected at 150 base pairs and processed for sequencing onto a single Illumina genome analyzer channel using the procedures recommended by the manufacturer.

### NGS library preparation for ChIP-seq

Approximately 10 ng of immunoprecipitated DNA from each sample were used for library preparation using the TruSeq ChIP Sample Prep protocol (Illumina) as described in the data sheet (Illumina). First, DNA was end-repaired to remove 3′ and 5′ overhangs and subsequently adenylated on 3′ ends and adapter-ligated. After purification and PCR amplification for 18 cycles, samples were pooled (6-plex). The final multiplexed libraries were quantified, and 10 pm of final libraries were subsequently sequenced on a HiSeq1000 (Illumina) using the paired end protocol and 50 cycles each.

### Structural studies of ATAD2 bromodomain

Apo crystals of ATAD2 were obtained by sitting drop vapour diffusion method using the condition previously described (Filippakopoulos et al., 2012). Soaking experiments were performed in a drop containing stabilizing solution (PEG 3350, Bis-Tris, pH 5.5, ammonium phosphate) supplemented with 6 mM histone peptide and 25% ethylene glycol for 2 h. The soaked crystals were flash-cooled in liquid nitrogen, and diffraction data were collected in-house on Rigaku FR-E superbright source. Data were processed using MOSFLM (Leslie, 2006) and subsequently scaled with SCALA from CCP4 suite (Collaborative Computational Project, Number 4, 1994). Structure solution was achieved by molecular replacement using PHASER program (McCoy et al., 2005) and the published ATAD2 apo structure (Filippakopoulos et al., 2012). Model rebuilding alternated with refinement was performed in COOT (Emsley and Cowtan, 2004) and REFMAC (Murshudov et al., 1997), respectively. The data collection and refinement statistics are summarized in Table 2.

**Table 2:** Data collection and refinement statistics.

Complexes (PDB IDs)	ATAD2-H4K5 (4QUU)	ATAD2-H4K12 (4QUT)
Data collection		
Beamline	Rigaku FR-E Superbright	Rigaku FR-E Superbright
Wavelength (Å)	1.5418	1.5418
Resolution^a^ (Å)	34.44-1.80 (1.90-1.80)	34.34-1.70 (1.79-1.70)
Spacegroup	*P*6_5_22	*P*6_5_22
Cell dimensions	*a* = *b* = 79.1*, c* = 139.0 Å	*a* = *b* = 79.0, *c* = 139.0 Å
	*α* = *β* = 90.0°, *γ* = 120.0°	*α* = *β* = 90.0°, *γ* = 120.0°
No. of unique reflections^a^	24588 (3483)	28321 (3895)
Completeness^a^ (%)	100.0 (100.0)	98.2 (94.9)
*I*/*σI*^a^	16.2 (2.4)	15.8 (2.4)
*R*_merge_^a^ (%)	0.090 (0.894)	0.070 (0.665)
Redundancy^a^	9.7 (9.9)	6.7 (5.9)
Refinement		
No. of atoms in refinement (P/His/O)^b^	1165/35/274	1178/32/314
*R*_fact_ (%)	16.9	16.2
*R*_free_ (%)	20	18
*B*_f_ (P/His/O)^b^ (Å^2^)	25/42/43	21/35/40
rms deviation bond^c^ (Å)	0.016	0.016
rms deviation angle^c^ (°)	1.5	1.5

^a^Values in brackets show the statistics for the highest resolution shells.

^b^P/His/O indicate protein, histone peptide, and others (water and other molecules), respectively.

^c^rms indicates root-mean-square.

## Supplementary material

Supplementary material is available at *Journal of Molecular Cell Biology* online.

## Funding

This project was performed in the frame of a network supported by INCa (http://www.e-cancer.fr/) involving S.K., M.G., and C.P. laboratories. S.K. also acknowledges the support of ANR (http://www.agence-nationale-recherche.fr/) EpiSperm2 project. Y.M. is a recipient of a post-doctoral fellowship from foundation ARC (http://www.fondation-arc.org/), and M.J. is supported by INCa (http://www.e-cancer.fr/). S.Kn. is supported by the SGC (http://www.thesgc.org/), a registered charity (number 1097737) that receives funds from AbbVie, Bayer, Boehringer Ingelheim, the Canada Foundation for Innovation, the Canadian Institutes for Health Research, Genome Canada, GlaxoSmithKline, Janssen, Lilly Canada, the Novartis Research Foundation, the Ontario Ministry of Economic Development and Innovation, Pfizer, Takeda, and the Wellcome Trust [092809/Z/10/Z]. A.C. is supported by the European Union FP7 Grant (http://ec.europa.eu/research/fp7/) No. 278568 ‘PRIMES’ (Protein interaction machines in oncogenic EGF receptor signalling).

## 

**Conflict of interest:** N.S. and D.G. are Boehringer Ingelheim employees.

## Supplementary Material

Supplementary Data
