# Supplementary material for: Atad2 is a generalist facilitator of chromatin dynamics in embryonic stem cells
Source: J Mol Cell Biol. 2016 Aug 19;8(4):349–62. doi: 10.1093/jmcb/mjv060 (PMC4991664; doi:10.1093/jmcb/mjv060)
Supplement: Supplementary Data [file supp_8_4_349__index.html]

Atad2 is a generalist facilitator of chromatin dynamics in embryonic stem cells — Atad2 is a generalist facilitator of chromatin dynamics in embryonic stem cells — Atad2 is a generalist facilitator of chromatin dynamics in embryonic stem cells — Supplementary Data 

# Atad2 is a generalist facilitator of chromatin dynamics in embryonic stem cells

## Supplementary Data

Supplementary Data

- Supplementary Data - Pdf file
