# Supplementary material for: Atad2 is a generalist facilitator of chromatin dynamics in embryonic stem cells
Source: J Mol Cell Biol. 2016 Aug 19;8(4):349–62. doi: 10.1093/jmcb/mjv060 (PMC4991664; doi:10.1093/jmcb/mjv060)
Supplement: Supplementary Data [file supp_mjv060_mjv060supp.pdf]

Supplementary Figures

Figure S1 (related to Figure 2D). Atad2 knockdown does not affect the expression of repeated sequences in ESCs.

The RNA-seq FastQ files were mapped on the genome using Bowtie. (a) Mapped repeats: a search for overlaps between mapped reads (BAM files) and UCSC mouse repeat masker, identified 1217 aligned repeats, whose read counts were similar between si-Atad2 and si-ctrl ES cells. (B-F) Unmapped repeats: files corresponding to unaligned reads Left (L) or Right (R) were processed through the web application http://compbio.med.harvard.edu/repeats/ and the results expressed as the log2 of the read counts corresponding to repeats elements in the standard analysis (b and c: uses reads that can be uniquely associated with a single repeat type) as well as in the conservative analysis (d and e: removes the reads associated with sequences not annotated as an instance of a known repeat type). Table f shows the total number of reads corresponding to each analysis. No difference was observed between si-Ctrl and si-Atad2 cells.

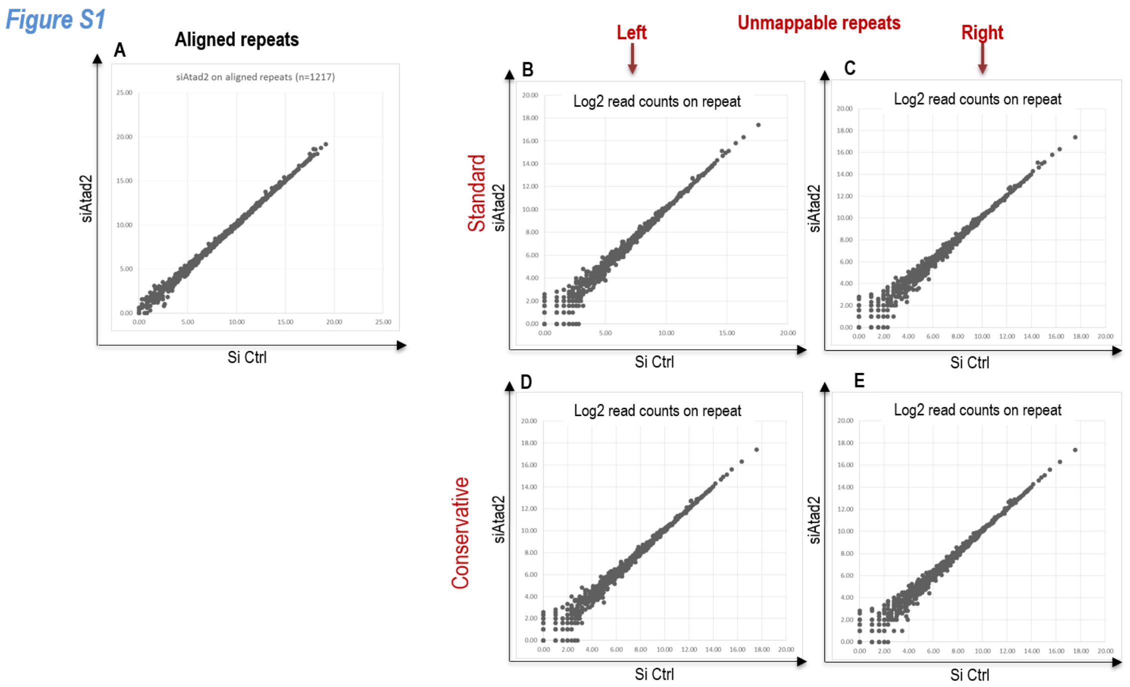

F	Repeats (left) siAtad2		Repeats (left) siCtrl		Repeats (right) SiAtad2		Repeats (right) siCtrl	
	Count	%	Count	%	Count	%	Count	%
Total tags	62316180	100%	62322804	100%	62316180	100%	62322804	100%
Mappable tags	41932990	67%	41954968	67%	41329389	66%	41329876	66%
Repeats (standard)	1055636	2%	1031644	2%	1026984	2%	1002629	2%
Repeats (conservative)	992781	2%	981032	2%	966427	2%	953727	2%

Figure S2 (related to Figure 3). Visualisation of Atad2-bound chromatin-associated proteins.

A higher magnification of the silver-stained gel shown in Figure 3B, is presented to better visualize the gel slices analysed by MS. The slices corresponding to the parallel IP (46C and $46C^{Tag}$) are numbered as indicated. The protein identified in the 46C gel slices (control) were removed from the list of proteins identified in the $46C^{tag}$ gel slices, which were then listed in Table S1A in different functional categories.

Figure S2

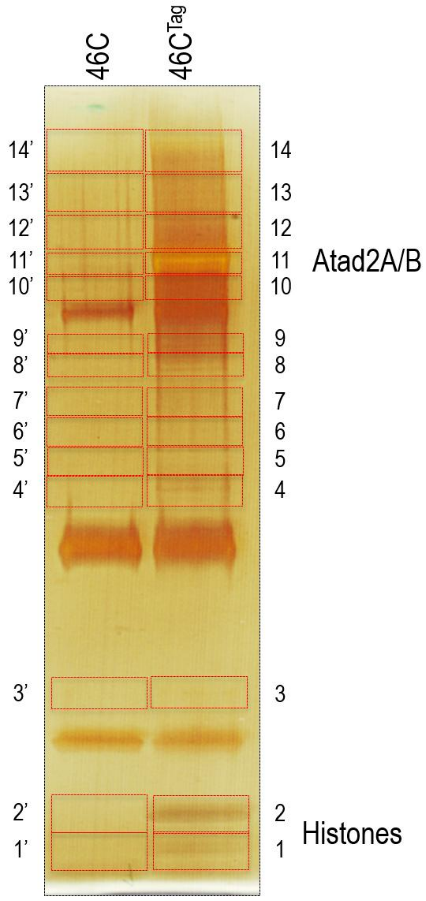

Figure S3 (related to Figure 3C). Comparative Atad2 and Yta7 ChIP-proteomics. Figure 3C (left) is shown next to the Yta7 ChIP-proteomics data extracted from data published by Lambert and colleagues (see reference Lambert et al., 2010, in the main text).

Figure S3

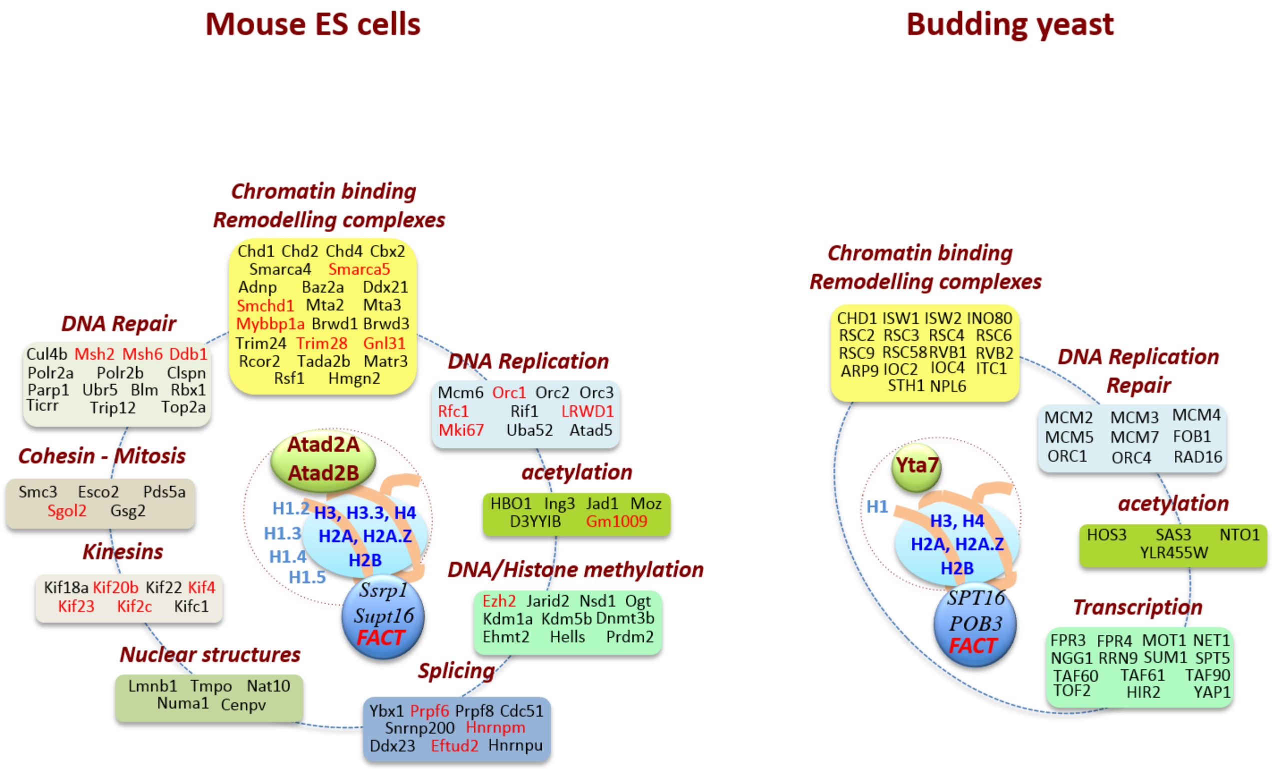

Figure S4 (related to Figure 4B). Characterization of acetylated histones co-purified with Atad2.

Annotated MS/MS spectra of the identified histone Kac peptides from silver stained gel in Figure 4B are shown.

(1)

H3 K79
EIAQDF<u>K</u>TDLR

MS/MS Fragmentation of **EIAQDFKTDLR**
Found in **gi|386772**, histone H3 [Homo sapiens]

Match to Query 6701: 1376.693208 from(689.353880,2+) intensity(33260.8125)
Title: File4012 Spectrum9839 scans: 10905
Data file 2014-4-22-TAG-4-ZY.mgf

Click mouse within plot area to zoom in by factor of two about that point
Or, Plot from 100 to 1300 Da Full range
Label all possible matches ○ Label matches used for scoring ◉

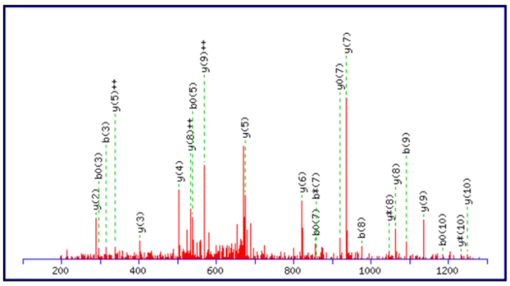

Monoisotopic mass of neutral peptide Mr(calc): 1376.6936
Variable modifications:
K7 : Acetyl (K)
Ions Score: 56 **Expect:** 2.7e-006
Matches : 23/110 fragment ions using 44 most intense peaks (help)

#	b	b^{++}	b*	b^{*++}	b^{0}	b^{0++}	Seq.	y	y^{++}	y*	y^{*++}	y^{0}	y^{0++}	#
1	130.0499	65.5286			112.0393	56.5233	E							11
2	243.1339	122.0706			225.1234	113.0653	I	***1248.6583***	624.8328	1231.6317	616.3195	1230.6477	615.8275	10
3	**314.1710**	157.5892			296.1605	148.5839	A	***1135.5742***	568.2907	1118.5477	559.7775	1117.5636	559.2855	9
4	442.2296	221.6185	425.2031	213.1052	424.2191	212.6132	Q	***1064.5371***	532.7722	1047.5106	524.2589	1046.5265	523.7669	8
5	557.2566	279.1319	540.2300	270.6186	539.2460	270.1266	D	***936.4785***	468.7429	919.4520	460.2296	918.4680	459.7376	7
6	704.3250	352.6661	687.2984	344.1529	686.3144	343.6608	F	***821.4516***	411.2294	804.4250	402.7162	803.4410	402.2241	6
7	874.4305	437.7189	857.4040	429.2056	856.4199	428.7136	K	***674.3832***	337.6952	657.3566	329.1819	656.3726	328.6899	5
8	**975.4782**	488.2427	958.4516	479.7295	957.4676	479.2375	T	***504.2776***	252.6425	487.2511	244.1292	486.2671	243.6372	4
9	**1090.5051**	545.7562	1073.4786	537.2429	1072.4946	536.7509	D	***403.2300***	202.1186	386.2034	193.6053	385.2194	193.1133	3
10	1203.5892	602.2982	1186.5626	593.7850	1185.5786	593.2930	L	***288.2030***	144.6051	271.1765	136.0919			2
11							R	175.1190	88.0631	158.0924	79.5498			1

(2)

MS/MS Fragmentation of KQLATKAAR
Found in gi|386772, histone H3 [Homo sapiens]

H3 K18 K23
KQLATKAAR

Match to Query 3535: 1069.623988 from(535.819270,2+) intensity(787636.2500)
Title: File4012 Spectrum3718 scans: 4376
Data file 2014-4-22-TAG-4-ZY.mgf

Click mouse within plot area to zoom in by factor of two about that point
Or, Plot from 150 to 950 Da Full range
Label all possible matches ○ Label matches used for scoring ●

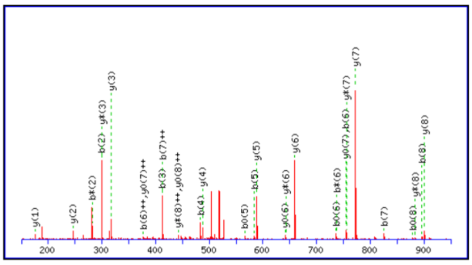


```
Monoisotopic mass of neutral peptide Mr(calc): 1069.6243
Variable modifications:
K1      : Acetyl (K)
K6      : Acetyl (K)
Ions Score: 50  Expect: 1e-005
Matches : 31/80 fragment ions using 56 most intense peaks   (help)
```

#	b	b^{++}	b*	b^{*++}	b^0	b^{0++}	Seq.	y	y^{++}	y*	y^{*++}	y^0	y^{0++}	#
1	171.1128	86.0600	154.0863	77.5468			K							9
2	**299.1714**	150.0893	282.1448	141.5761			Q	*900.5261*	450.7667	883.4996	442.2534	882.5156	441.7614	8
3	**412.2554**	206.6314	395.2289	198.1181			L	*772.4676*	386.7374	755.4410	378.2241	754.4570	377.7321	7
4	**483.2926**	242.1499	466.2660	233.6366			A	*659.3835*	330.1954	642.3570	321.6821	641.3729	321.1901	6
5	**584.3402**	292.6738	567.3137	284.1605	566.3297	283.6685	T	*588.3464*	294.6768	571.3198	286.1636	570.3358	285.6715	5
6	**754.4458**	377.7265	737.4192	369.2132	736.4352	368.7212	K	*487.2987*	244.1530	470.2722	235.6397			4
7	**825.4829**	413.2451	808.4563	404.7318	807.4723	404.2398	A	*317.1932*	159.1002	300.1666	150.5870			3
8	**896.5200**	448.7636	879.4934	440.2504	878.5094	439.7584	A	*246.1561*	123.5817	229.1295	115.0684			2
9							R	*175.1190*	88.0631	158.0924	79.5498			1

(3)

MS/MS Fragmentation of KSTGGKAPR
Found in gi|386772, histone H3 [Homo sapiens]

H3 K9 K14
KSTGGKAPR

Match to Query 2768: 984.535488 from(493.275020,2+) intensity(113088.2813)
Title: File4012 Spectrum1601 scans: 1998
Data file 2014-4-22-TAG-4-ZY.mgf

Click mouse within plot area to zoom in by factor of two about that point
Or, Plot from 100 to 1000 Da Full range
Label all possible matches ○ Label matches used for scoring ●

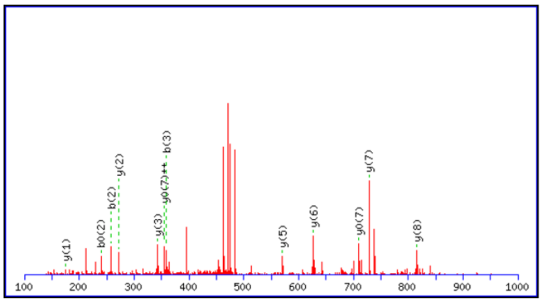


```
Monoisotopic mass of neutral peptide Mr(calc): 984.5352
Variable modifications:
K1      : Acetyl (K)
K6      : Acetyl (K)
Ions Score: 41  Expect: 7.7e-005
Matches : 12/82 fragment ions using 33 most intense peaks   (help)
```

#	b	b^{++}	b*	b^{*++}	b^0	b^{0++}	Seq.	y	y^{++}	y*	y^{*++}	y^0	y^{0++}	#
1	171.1128	86.0600	154.0863	77.5468			K							9
2	**258.1448**	129.5761	241.1183	121.0628	240.1343	120.5708	S	*815.4370*	408.2221	798.4104	399.7089	797.4264	399.2169	8
3	**359.1925**	180.0999	342.1660	171.5866	341.1819	171.0946	T	*728.4050*	364.7061	711.3784	356.1928	710.3944	355.7008	7
4	416.2140	208.6106	399.1874	200.0974	398.2034	199.6053	G	*627.3573*	314.1823	610.3307	305.6690			6
5	473.2354	237.1214	456.2089	228.6081	455.2249	228.1161	G	*570.3358*	285.6715	553.3093	277.1583			5
6	643.3410	322.1741	626.3144	313.6608	625.3304	313.1688	K	513.3144	257.1608	496.2878	248.6475			4
7	714.3781	357.6927	697.3515	349.1794	696.3675	348.6874	A	*343.2088*	172.1081	326.1823	163.5948			3
8	811.4308	406.2191	794.4043	397.7058	793.4203	397.2138	P	*272.1717*	136.5895	255.1452	128.0762			2
9							R	*175.1190*	88.0631	158.0924	79.5498			1

(4)

MS/MS Fragmentation of **GKGGKGLGKGGAK**
Found in **gi|28173560**, histone H4 [Homo sapiens]

H4 K5 K8 K12
GKGGKGLGKGGAK

Match to Query 5152: 1239.691988 from(620.853270,2+) intensity(49482.3711)
Title: File4012 Spectrum3719 scans: 4377
Data file 2014-4-22-TAG-4-ZY.mgf

Click mouse within plot area to zoom in by factor of two about that point
Or, Plot from 200 to 1150 Da Full range
Label all possible matches ○ Label matches used for scoring ◉

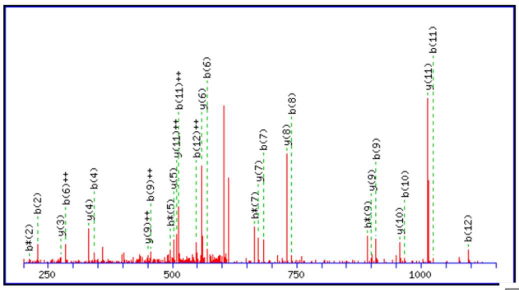


```
Monoisotopic mass of neutral peptide Mr(calc): 1239.6935
Variable modifications:
K2     : Acetyl (K)
K5     : Acetyl (K)
K9     : Acetyl (K)
Ions Score: 57   Expect: 1.9e-006
Matches : 30/94 fragment ions using 76 most intense peaks   (help)
```

#	b	b++	b*	b*++	Seq.	y	y++	y*	y*++	#
1	58.0287	29.5180			G					13
2	***228.1343***	114.5708	211.1077	106.0575	K	1183.6794	592.3433	1166.6528	583.8300	12
3	***285.1557***	143.0815	268.1292	134.5682	G	**1013.5738**	507.2905	996.5473	498.7773	11
4	***342.1772***	171.5922	325.1506	163.0790	G	**956.5524**	478.7798	939.5258	470.2665	10
5	***512.2827***	256.6450	495.2562	248.1317	K	**899.5309**	450.2691	882.5043	441.7558	9
6	***569.3042***	**285.1557**	552.2776	276.6425	G	**729.4254**	365.2163	712.3988	356.7030	8
7	***682.3883***	341.6978	665.3617	333.1845	L	**672.4039**	336.7056	655.3774	328.1923	7
8	***739.4097***	370.2085	722.3832	361.6952	G	**559.3198**	280.1636	542.2933	271.6503	6
9	***909.5152***	**455.2613**	892.4887	446.7480	K	**502.2984**	251.6528	485.2718	243.1396	5
10	***966.5367***	483.7720	949.5102	475.2587	G	**332.1928**	166.6001	315.1663	158.0868	4
11	***1023.5582***	**512.2827**	1006.5316	503.7694	G	**275.1714**	138.0893	258.1448	129.5761	3
12	***1094.5953***	**547.8013**	1077.5687	539.2880	A	218.1499	109.5786	201.1234	101.0653	2
13					K	147.1128	74.0600	130.0863	65.5468	1

(5)

MS/MS Fragmentation of **GKGGKGLGKGGAKR**
Found in **gi|28173560**, histone H4 [Homo sapiens]

H4 K5 K8 K12 K16
GKGGKGLGKGGAKR

Match to Query 7433: 1437.807228 from(719.910890,2+) intensity(56822.3945)
Title: File4012 Spectrum4387 scans: 5100
Data file 2014-4-22-TAG-4-ZY.mgf

Click mouse within plot area to zoom in by factor of two about that point
Or, Plot from 200 to 1500 Da Full range
Label all possible matches ○ Label matches used for scoring ◉

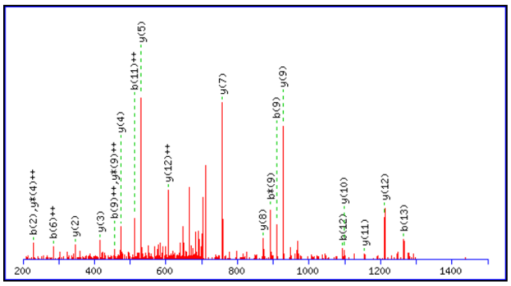


```
Monoisotopic mass of neutral peptide Mr(calc): 1437.8052
Variable modifications:
K2     : Acetyl (K)
K5     : Acetyl (K)
K9     : Acetyl (K)
K13    : Acetyl (K)
Ions Score: 72   Expect: 5.6e-008
Matches : 23/102 fragment ions using 33 most intense peaks   (help)
```

#	b	b++	b*	b*++	Seq.	y	y++	y*	y*++	#
1	58.0287	29.5180			G					14
2	**228.1343**	114.5708	211.1077	106.0575	K	1381.7910	691.3992	1364.7645	682.8859	13
3	**285.1557**	143.0815	268.1292	134.5682	G	***1211.6855***	606.3464	1194.6589	597.8331	12
4	342.1772	171.5922	325.1506	163.0790	G	***1154.6640***	577.8357	1137.6375	569.3224	11
5	**512.2827**	256.6450	495.2562	248.1317	K	***1097.6426***	549.3249	1080.6160	540.8116	10
6	569.3042	**285.1557**	552.2776	276.6425	G	***927.5370***	464.2722	910.5105	455.7589	9
7	682.3883	341.6978	665.3617	333.1845	L	***870.5156***	435.7614	853.4890	427.2482	8
8	739.4097	370.2085	722.3832	361.6952	G	***757.4315***	379.2194	740.4050	370.7061	7
9	**909.5152**	**455.2613**	892.4887	446.7480	K	700.4101	350.7087	683.3835	342.1954	6
10	966.5367	483.7720	949.5102	475.2587	G	***530.3045***	265.6559	513.2780	257.1426	5
11	1023.5582	**512.2827**	1006.5316	503.7694	G	***473.2831***	237.1452	456.2565	228.6319	4
12	**1094.5953**	547.8013	1077.5687	539.2880	A	***416.2616***	208.6344	399.2350	200.1212	3
13	**1264.7008**	632.8540	1247.6743	624.3408	K	***345.2245***	173.1159	328.1979	164.6026	2
14					R	175.1190	88.0631	158.0924	79.5498	1

Figure S5 (related to Figure 4D). Y1021F mutation inactivates the ability of ATAD2 bromodomain to bind acetylated histones.

ATAD2 bromodomain from wild type or the Y1021F mutant was amplified and cloned in a bacterial expression vector in fusion with GST. After the induction of bacteria with IPTG the corresponding bacterial extracts were used to perform a pull-down assay using biotinylated unmodified or tetra-acetylated H4 tail peptides immobilized on steptavidin beads. Pull-downed protein were then detected using anti-GST antibody.

Figure S5

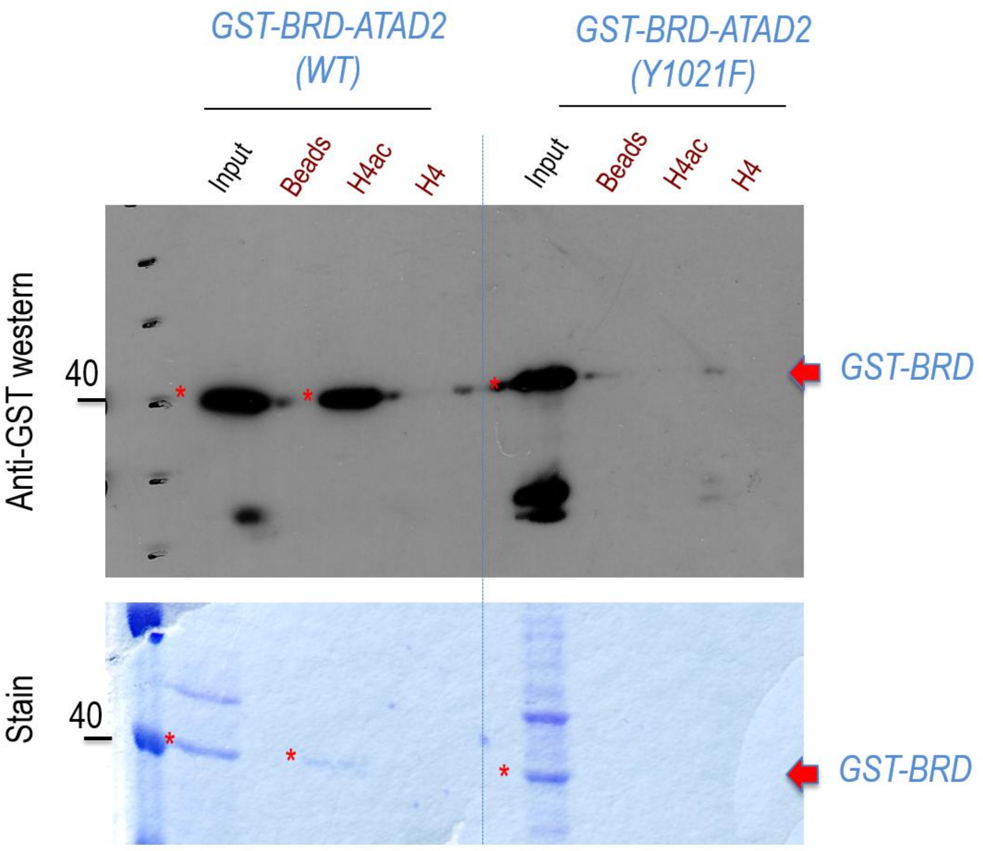

* Indicates the position of GST-BRD in the extracts and after pull down

Figure S6 (related to Figure 4). Atad2 is enriched in acetylated chromatin regions.

Aligned raw reads from ChIPseq data in ES cells from Ren's lab were recovered from Encode (http://genome.ucsc.edu/) for H3K9ac and H3K27ac (UCSC references: wgEncodeEM003176 and wgEncodeEM002497 respectively) and used for peak calling to identify the regions enriched in acetylated histones. These data were crossed with the Atad2 ChIP seq data from both experiments (Exp1 and Exp2) and the Atad2 reads numbers were compared between the non-acetylated regions (-) and the acetylated regions (+), within the Atad2-bound overlapping regions.

Figure S6

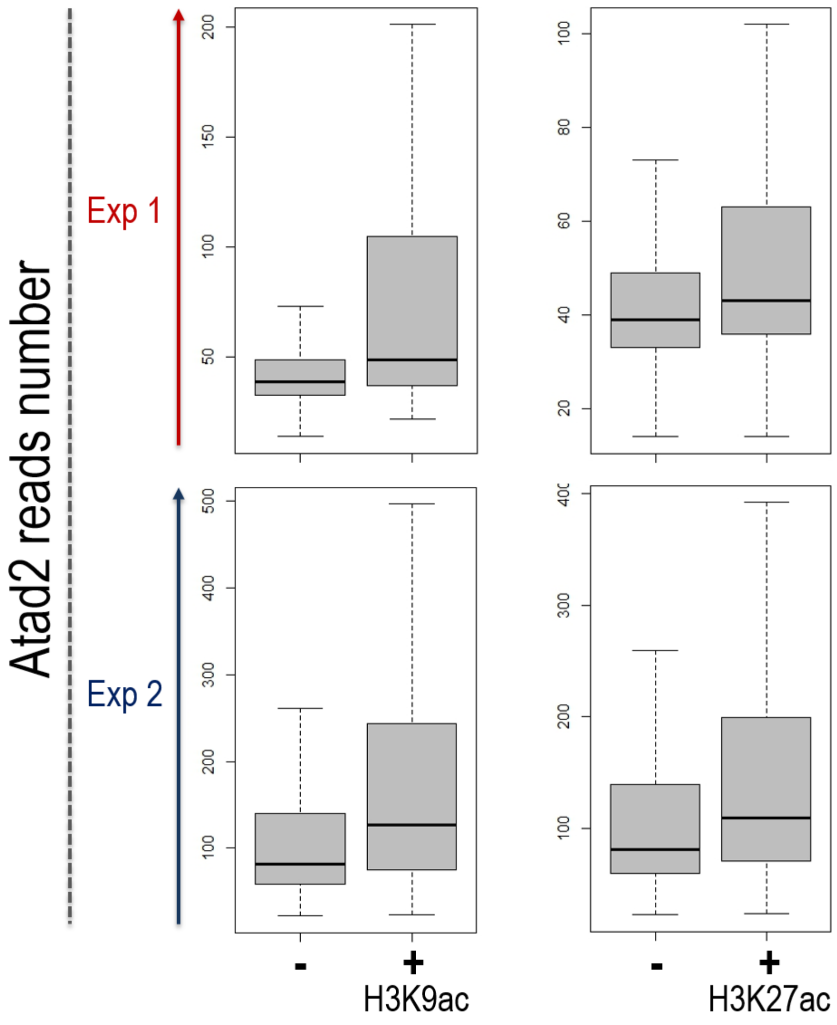

Supplementary Tables

Table S1 (related to Figure 3C, Figure S2 and Figure 4B). Identification of proteins co-purified with chromatin-bound Atad2. (**A**) List of proteins identified in gel slices indicated in Figure S2 ($C46^{Tag}$ gel slices) as Atad2-nucleosome associated after removing proteins found in the 46C-immunoprecipitated materials (Figure S2, C46 slices) or with a score cut-off below 30. The list is organized as a function of the proteins' functional annotations as indicated. **(B)** The list of the identified proteins corresponding to histone bands on the silver-stained gel shown in Figure 4B. (**C**) The list of acetylated histone spectra (related to Figure 4B).

Table S1A

Index	CPDB_groups	prot_acc	GeneSymbol	prot_desc	prot_score (tag or tag-46C)	Band	prot_hit_num	prot_MW	ScoreTag/46C
1	**Atad2**	**G3X963**	**Atad2**	**ATPase family AAA domain-containing protein 2 OS=Mus musculus GN=Atad2 PE=4 SV=1**	**16130**	**Tag11**	**1**	**155188**	**171**
2	**Atad2**	**E9Q166**	**Atad2b**	**Protein Atad2b OS=Mus musculus GN=Atad2b PE=2 SV=1**	**12132**	**Tag11**	**2**	**164621**	**90**
3	Chromatin binding/remodelling and complexes	A2BDX0	Adnp	Activity-dependent neuroprotector homeobox protein OS=Mus musculus GN=Adnp PE=3 SV=1	33	Tag10	49	124229	33
4	Chromatin binding/remodelling and complexes	F8VPM0	Baz2a	Bromodomain adjacent to zinc finger domain protein 2A OS=Mus musculus GN=Baz2a PE=2 SV=1	280	Tag12	11	209275	280
5	Chromatin binding/remodelling and complexes	Q921C3	Brwd1	Bromodomain and WD repeat-containing protein 1 OS=Mus musculus GN=Brwd1 PE=1 SV=2	214	Tag13	12	259066	214
6	Chromatin binding/remodelling and complexes	A2AHJ4	Brwd3	Bromodomain and WD repeat-containing protein 3 OS=Mus musculus GN=Brwd3 PE=2 SV=1	104	Tag12	23	202816	104
7	Chromatin binding/remodelling and complexes	P30658	Cbx2	Chromobox protein homolog 2 OS=Mus musculus GN=Cbx2 PE=1 SV=2	60	Tag5	81	54885	60
8	Chromatin binding/remodelling and complexes	P40201	Chd1	Chromodomain-helicase-DNA-binding protein 1 OS=Mus musculus GN=Chd1 PE=1 SV=3	67	Tag12	29	196264	67
9	Chromatin binding/remodelling and complexes	E9PZM4	Chd2	Protein Chd2 OS=Mus musculus GN=Chd2 PE=2 SV=1	62	Tag12	32	210674	62
10	Chromatin binding/remodelling and complexes	Q6PDQ2	Chd4	Chromodomain-helicase-DNA-binding protein 4 OS=Mus musculus GN=Chd4 PE=1 SV=1	390	Tag12	6	217614	390
11	Chromatin binding/remodelling and complexes	Q9JIK5	Ddx21	Nucleolar RNA helicase 2 OS=Mus musculus GN=Ddx21 PE=1 SV=3	76	Tag9	23	93493	76
12	Chromatin binding/remodelling and complexes	Q6PGG6	Gnl3l	Guanine nucleotide-binding protein-like 3-like protein OS=Mus musculus GN=Gnl3l PE=1 SV=1	170	Tag5	31	65153	170
13	Chromatin binding/remodelling and complexes	A3KGL9	Hmgn2	Non-histone chromosomal protein HMG-17 OS=Mus musculus GN=Hmgn2 PE=2 SV=1	38	Tag13	42	9720	38
14	Chromatin binding/remodelling and complexes	Q8K310	Matr3	Matrin-3 OS=Mus musculus GN=Matr3 PE=1 SV=1	88	Tag9	20	94572	88
15	Chromatin binding/remodelling and complexes	Q9R190	Mta2	Metastasis-associated protein MTA2 OS=Mus musculus GN=Mta2 PE=1 SV=1	38	Tag6	24	74983	38
16	Chromatin binding/remodelling and complexes	Q3U3A7	Mta3	Metastasis-associated protein MTA3 OS=Mus musculus GN=Mta3 PE=2 SV=1	33	Tag4	101	58330	33
17	Chromatin binding/remodelling and complexes	Q7TPV4	Mybbp1a	Myb-binding protein 1A OS=Mus musculus GN=Mybbp1a PE=1 SV=2	191	Tag10	14	151942	191
18	Chromatin binding/remodelling and complexes	Q8C796	Rcor2	REST corepressor 2 OS=Mus musculus GN=Rcor2 PE=2 SV=1	57	Tag5	86	57872	57
19	Chromatin binding/remodelling and complexes	E9PWW9	Rsf1	Protein Rsf1 OS=Mus musculus GN=Rsf1 PE=2 SV=1	304	Tag12	9	160525	304
20	Chromatin binding/remodelling and complexes	G3UX35	Smarca4	Transcription activator BRG1 (Fragment) OS=Mus musculus GN=Smarca4 PE=4 SV=1	191	Tag10	14	143529	191
21	Chromatin binding/remodelling and complexes	Q91ZW3	Smarca5	SWI/SNF-related matrix-associated actin-dependent regulator of chromatin subfamily A member 5 O	147	Tag10	17	121550	147
22	Chromatin binding/remodelling and complexes	Q6P5D8	Smchd1	Structural maintenance of chromosomes flexible hinge domain-containing protein 1 OS=Mus musculu	30	Tag12	49	225506	30
23	Chromatin binding/remodelling and complexes	D3YW26	Tada2b	Protein Tada2b OS=Mus musculus GN=Tada2b PE=2 SV=1	39	Tag1	44	39977	39
24	Chromatin binding/remodelling and complexes	Q64127; E	Trim24	Transcription intermediary factor 1-alpha OS=Mus musculus GN=Trim24 PE=1 SV=1	91	Tag10	23	116583	91
25	Chromatin binding/remodelling and complexes	Q62318	Trim28	Transcription intermediary factor 1-beta OS=Mus musculus GN=Trim28 PE=1 SV=3	330	Tag8	11	88791	330
26	Fact	A2AW05	Ssrp1	FACT complex subunit SSRP1 (Fragment) OS=Mus musculus GN=Ssrp1 PE=2 SV=1	84	Tag7	47	71484	84
27	Fact	G3X956	Supt16	Protein Supt16 OS=Mus musculus GN=Supt16 PE=4 SV=1	324	Tag10	11	119763	324
28	Cohesins	Q8CIB9	Esco2	N-acetyltransferase ESCO2 OS=Mus musculus GN=Esco2 PE=2 SV=3	178	Tag5	28	67231	178
29	Cohesins	Q9Z0R0	Gsg2	Serine/threonine-protein kinase haspin OS=Mus musculus GN=Gsg2 PE=1 SV=3	56	Tag7	73	84129	56
30	Cohesins	Q6A026	Pds5a	Sister chromatid cohesion protein PDS5 homolog A OS=Mus musculus GN=Pds5a PE=3 SV=3	101	Tag10	21	150232	101
31	Cohesins	Q7TSY8	Sgol2	Shugoshin-like 2 OS=Mus musculus GN=Sgol2 PE=1 SV=1	643	Tag10	4	130196	643
32	Cohesins	Q9CW03	Smc3	Structural maintenance of chromosomes protein 3 OS=Mus musculus GN=Smc3 PE=1 SV=2	73	Tag10	26	141468	73
33	Histone acetylation	D3YUP8	Ing3	Inhibitor of growth protein 3 OS=Mus musculus GN=Ing3 PE=2 SV=1	37	Tag1	45	45528	37
34	Histone acetylation	Q8BRB7	Kat6b	Histone acetyltransferase KAT6B OS=Mus musculus GN=Kat6b PE=2 SV=3	290	Tag12	10	208394	290
35	Histone acetylation	Q5SVQ0	Kat7	Histone acetyltransferase KAT7 OS=Mus musculus GN=Kat7 PE=2 SV=1	80	Tag6	15	70597	80
36	Histone acetylation	Q6ZPI0	Phf17	Protein Jade-1 OS=Mus musculus GN=Phf17 PE=1 SV=2	157	Tag8	16	93838	157
37	Histone acetylation	D3YYI8	Gm10093	Histone deacetylase OS=Mus musculus GN=Gm10093 PE=3 SV=1	36	Tag4	95	55013	36
38	Histone methylation	Q60848	Hells	Lymphocyte-specific helicase OS=Mus musculus GN=Hells PE=1 SV=2	65	Tag8	34	95065	65
39	Histone methylation	Q8CGY8	Ogt	UDP-N-acetylglucosamine--peptide N-acetylglucosaminyltransferase 110 kDa subunit OS=Mus mus	44	Tag9	40	116877	44
40	Histone methylation	Q3KR45	Dnmt3b	DNA (cytosine-5)-methyltransferase 3B OS=Mus musculus GN=Dnmt3b PE=2 SV=1	53	Tag9	33	97294	53
41	Histone methylation	A2CG76	Ehmt2	Histone-lysine N-methyltransferase EHMT2 OS=Mus musculus GN=Ehmt2 PE=2 SV=1	35	Tag10	43	134602	35
42	Histone methylation	D3Z774	Ezh2	Histone-lysine N-methyltransferase EZH2 OS=Mus musculus GN=Ezh2 PE=2 SV=1	63	Tag7	66	80945	63
43	Histone methylation	Q62315	Jarid2	Protein Jumonji OS=Mus musculus GN=Jarid2 PE=1 SV=1	206	Tag10	13	137359	206
44	Histone methylation	A3KG93	Kdm1a	Lysine-specific histone demethylase 1A OS=Mus musculus GN=Kdm1a PE=2 SV=1	69	Tag9	25	94600	69
45	Histone methylation	Q80Y84	Kdm5b	Lysine-specific demethylase 5B OS=Mus musculus GN=Kdm5b PE=1 SV=1	110	Tag11	51	175442	110
46	Histone methylation	E9QAE4	Nsd1	Histone-lysine N-methyltransferase, H3 lysine-36 and H4 lysine-20-specific OS=Mus musculus GN=	42	Tag13	40	296178	42
47	Histone methylation	A2A7B5	Prdm2	Protein Prdm2 OS=Mus musculus GN=Prdm2 PE=2 SV=1	49	Tag12	36	187087	49
48	Histones	P0C0S6	H2afz	Histone H2A.Z OS=Mus musculus GN=H2afz PE=1 SV=2	361	Tag2	9	13545	7
49	Histones	F8WI35	H3f3a	Histone H3 OS=Mus musculus GN=H3f3a PE=2 SV=1	114	Tag1	19	15189	114
50	Histones	P02301	H3f3c	Histone H3.3C OS=Mus musculus GN=H3f3c PE=1 SV=3	678	Tag2	8	15306	678
51	Histones	P43276	Hist1h1b	Histone H1.5 OS=Mus musculus GN=Hist1h1b PE=1 SV=2	46	Tag7	82	22562	46
52	Histones	P15864	Hist1h1c	Histone H1.2 OS=Mus musculus GN=Hist1h1c PE=1 SV=2	84	Tag9	21	21254	84
53	Histones	P43277	Hist1h1d	Histone H1.3 OS=Mus musculus GN=Hist1h1d PE=1 SV=2	113	Tag5	44	22086	113
54	Histones	P43274	Hist1h1e	Histone H1.4 OS=Mus musculus GN=Hist1h1e PE=1 SV=2	107	Tag5	48	21964	107
55	Histones	P70696	Hist1h2ba	Histone H2B type 1-A OS=Mus musculus GN=Hist1h2ba PE=1 SV=3	1144	Tag2	5	14228	1144
56	Histones	Q6ZWY9	Hist1h2bc	Histone H2B type 1-C/E/G OS=Mus musculus GN=Hist1h2bc PE=1 SV=3	1667	Tag2	2	13898	17
57	Histones	P10853	Hist1h2bf	Histone H2B type 1-F/J/L OS=Mus musculus GN=Hist1h2bf PE=1 SV=2	1756	Tag2	3	13928	1756
58	Histones	Q8CGP1	Hist1h2bk	Histone H2B type 1-K OS=Mus musculus GN=Hist1h2bk PE=1 SV=3	1701	Tag2	4	13912	1701
59	Histones	P62806	Hist1h4a	Histone H4 OS=Mus musculus GN=Hist1h4a PE=1 SV=2	2067	Tag1	1	11360	4
60	Histones	Q6GSS7	Hist2h2aa1	Histone H2A type 2-A OS=Mus musculus GN=Hist2h2aa1 PE=1 SV=3	701	Tag2	7	14087	12
61	Histones	Q64522	Hist2h2ab	Histone H2A type 2-B OS=Mus musculus GN=Hist2h2ab PE=1 SV=3	99	Tag1	22	14005	99
62	Histones	Q64525	Hist2h2bb	Histone H2B type 2-B OS=Mus musculus GN=Hist2h2bb PE=1 SV=3	1786	Tag2	1	13912	1786
63	Histones	Q8BFU2	Hist3h2a	Histone H2A type 3 OS=Mus musculus GN=Hist3h2a PE=1 SV=3	946	Tag2	6	14113	946
64	Kinesins	Q91WD7	Kif18a	Kinesin-like protein KIF18A OS=Mus musculus GN=Kif18a PE=2 SV=1	49	Tag8	46	100871	49
65	Kinesins	Q80WE4	Kif20b	Kinesin-like protein KIF20B OS=Mus musculus GN=Kif20b PE=1 SV=3	260	Tag12	12	203389	260
66	Kinesins	Q3V300	Kif22	Kinesin-like protein KIF22 OS=Mus musculus GN=Kif22 PE=2 SV=2	126	Tag6	9	73145	126
67	Kinesins	E9Q5G3	Kif23	Protein Kif23 OS=Mus musculus GN=Kif23 PE=2 SV=1	53	Tag9	32	108707	53
68	Kinesins	Q922S8	Kif2c	Kinesin-like protein KIF2C OS=Mus musculus GN=Kif2c PE=1 SV=1	77	Tag7	55	81034	77
69	Kinesins	P33174	Kif4	Chromosome-associated kinesin KIF4 OS=Mus musculus GN=Kif4 PE=1 SV=3	507	Tag10	6	139432	507
70	Kinesins	Q9QWT9	Kifc1	Kinesin-like protein KIFC1 OS=Mus musculus GN=Kifc1 PE=1 SV=2	115	Tag6	11	74107	115
71	NuclearStructure	P14733	Lmnb1	Lamin-B1 OS=Mus musculus GN=Lmnb1 PE=1 SV=3	36	Tag13	44	66745	36
72	NuclearStructure	Q61033	Tmpo	Lamina-associated polypeptide 2, isoforms alpha/zeta OS=Mus musculus GN=Tmpo PE=1 SV=4	47	Tag7	81	75122	47
73	NuclearStructure	Q8K224	Nat10	N-acetyltransferase 10 OS=Mus musculus GN=Nat10 PE=2 SV=1	212	Tag9	12	115346	212
74	NuclearStructure	E9Q7G0	Numa1	Protein Numa1 OS=Mus musculus GN=Numa1 PE=2 SV=1	2272	Tag12	2	235487	2272
75	NuclearStructure	Q9CXS4	Cenpv	Centromere protein V OS=Mus musculus GN=Cenpv PE=2 SV=2	173	Tag3	6	27524	173
76	Sphase & replication	E9PVX6	Mki67	Protein Mki67 OS=Mus musculus GN=Mki67 PE=2 SV=1	372	Tag13	7	350650	372
77	Sphase & replication	Q4QY64	Atad5	ATPase family AAA domain-containing protein 5 OS=Mus musculus GN=Atad5 PE=1 SV=1	326	Tag12	8	203783	326
78	Sphase & replication	Q8BUI3	LRWD1	Leucine-rich repeat and WD repeat-containing protein 1 OS=Mus musculus GN=LRWD1 PE=2 SV=	444	Tag5	11	71549	444
79	Sphase & replication	P97311	Mcm6	DNA replication licensing factor MCM6 OS=Mus musculus GN=Mcm6 PE=1 SV=1	61	Tag8	37	92809	61
80	Sphase & replication	Q9Z1N2	Orc1	Origin recognition complex subunit 1 OS=Mus musculus GN=Orc1 PE=1 SV=2	414	Tag8	10	95043	414
81	Sphase & replication	Q60862	Orc2	Origin recognition complex subunit 2 OS=Mus musculus GN=Orc2 PE=1 SV=1	284	Tag5	16	65853	284
82	Sphase & replication	Q9JK30	Orc3	Origin recognition complex subunit 3 OS=Mus musculus GN=Orc3 PE=1 SV=1	139	Tag6	7	82289	139
83	Sphase & replication	G3UWX1	Rfc1	Replication factor C subunit 1 OS=Mus musculus GN=Rfc1 PE=2 SV=1	457	Tag10	8	125787	457
84	Sphase & replication	Q6PR54	Rif1	Telomere-associated protein RIF1 OS=Mus musculus GN=Rif1 PE=1 SV=2	744	Tag13	5	266063	744
85	Sphase & replication	E9Q9J0	Uba52	Ubiquitin-60S ribosomal protein L40 (Fragment) OS=Mus musculus GN=Uba52 PE=2 SV=1	180	Tag11	28	10907	180
86	Repair	E9PZ97	Blm	Bloom syndrome protein homolog OS=Mus musculus GN=Blm PE=2 SV=1	31	Tag11	98	158666	31
87	Repair	Q80YR7	Clspn	Claspin OS=Mus musculus GN=Clspn PE=2 SV=2	41	Tag12	38	146626	41
88	Repair	A2A432	Cul4b	Cullin-4B OS=Mus musculus GN=Cul4b PE=1 SV=1	245	Tag9	10	110630	245
89	Repair	Q3U1J4	Ddb1	DNA damage-binding protein 1 OS=Mus musculus GN=Ddb1 PE=1 SV=2	66	Tag9	26	126772	66
90	Repair	P43247	Msh2	DNA mismatch repair protein Msh2 OS=Mus musculus GN=Msh2 PE=2 SV=1	544	Tag9	4	104085	544
91	Repair	P54276	Msh6	DNA mismatch repair protein Msh6 OS=Mus musculus GN=Msh6 PE=1 SV=3	318	Tag11	16	150989	318
92	Repair	Q921K2	Parp1	Poly (ADP-ribose) polymerase family, member 1 OS=Mus musculus GN=Parp1 PE=2 SV=1	40	Tag9	42	112650	40
93	Repair	P08775	Polr2a	DNA-directed RNA polymerase II subunit RPB1 OS=Mus musculus GN=Polr2a PE=1 SV=3	77	Tag12	26	217039	77
94	Repair	Q8CFI7	Polr2b	DNA-directed RNA polymerase II subunit RPB2 OS=Mus musculus GN=Polr2b PE=2 SV=2	34	Tag10	47	133825	34
95	Repair	P62878	Rbx1	E3 ubiquitin-protein ligase RBX1 OS=Mus musculus GN=Rbx1 PE=1 SV=1	77	Tag1	32	12266	77
96	Repair	Q8BQ33	Ticrr	Treslin OS=Mus musculus GN=Ticrr PE=2 SV=2	79	Tag12	25	208205	79
97	Repair	Q01320	Top2a	DNA topoisomerase 2-alpha OS=Mus musculus GN=Top2a PE=1 SV=2	45	Tag11	80	172682	45
98	Repair	G5E870	Trip12	E3 ubiquitin-protein ligase TRIP12 OS=Mus musculus GN=Trip12 PE=1 SV=1	647	Tag12	5	223988	647
99	Repair	E9Q2H1	Ubr5	E3 ubiquitin-protein ligase UBR5 OS=Mus musculus GN=Ubr5 PE=2 SV=1	104	Tag13	18	308774	104

100	Splicing	Q6A068	Cdc5l	Cell division cycle 5-like protein OS=Mus musculus GN=Cdc5l PE=1 SV=2	38	Tag8	56	92133	38
101	Splicing	D3Z0M9	Ddx23	MCG18410, isoform CRA_a OS=Mus musculus GN=Ddx23 PE=3 SV=1	36	Tag8	58	95436	36
102	Splicing	A2AH85	Eftud2	116 kDa U5 small nuclear ribonucleoprotein component OS=Mus musculus GN=Eftud2 PE=4 SV=1	31	Tag9	45	109422	31
103	Splicing	B8JK32	Hnrnpm	Heterogeneous nuclear ribonucleoprotein M OS=Mus musculus GN=Hnrnpm PE=2 SV=1	227	Tag6	3	72022	227
104	Splicing	G3XA10	Hnrnpu	Heterogeneous nuclear ribonucleoprotein U OS=Mus musculus GN=Hnrnpu PE=4 SV=1	305	Tag9	7	86751	305
105	Splicing	Q91YR7	Prpf6	Pre-mRNA-processing factor 6 OS=Mus musculus GN=Prpf6 PE=2 SV=1	478	Tag9	5	106655	478
106	Splicing	Q99PV0	Prpf8	Pre-mRNA-processing-splicing factor 8 OS=Mus musculus GN=Prpf8 PE=1 SV=2	130	Tag12	19	273443	130
107	Splicing	Q6P4T2	Snrnp200	U5 small nuclear ribonucleoprotein 200 kDa helicase OS=Mus musculus GN=Snrnp200 PE=2 SV=1	341	Tag12	7	244392	341
108	Splicing	A2BGG7	Ybx1	Nuclease-sensitive element-binding protein 1 (Fragment) OS=Mus musculus GN=Ybx1 PE=2 SV=1	56	Tag4	62	24726	56
109	Ribosomal proteins	P47963	Rpl13	60S ribosomal protein L13 OS=Mus musculus GN=Rpl13 PE=2 SV=3	118	Tag3	9	24290	118
110	Ribosomal proteins	P62830	Rpl23	60S ribosomal protein L23 OS=Mus musculus GN=Rpl23 PE=1 SV=1	41	Tag2	25	14856	41
111	Ribosomal proteins	Q8BP67	Rpl24	60S ribosomal protein L24 OS=Mus musculus GN=Rpl24 PE=2 SV=2	48	Tag3	20	17768	48
112	Ribosomal proteins	P83882	Rpl36a	60S ribosomal protein L36a OS=Mus musculus GN=Rpl36a PE=2 SV=2	44	Tag2	24	12433	44
113	Ribosomal proteins	P61514	Rpl37a	60S ribosomal protein L37a OS=Mus musculus GN=Rpl37a PE=2 SV=2	51	Tag1	39	10268	51
114	Ribosomal proteins	P62918	Rpl8	60S ribosomal protein L8 OS=Mus musculus GN=Rpl8 PE=2 SV=2	55	Tag3	18	28007	55
115	Ribosomal proteins	P62264	Rps14	40S ribosomal protein S14 OS=Mus musculus GN=Rps14 PE=2 SV=3	47	Tag2	22	16263	47
116	Ribosomal proteins	G3UZW2	Rps18	40S ribosomal protein S18 OS=Mus musculus GN=Rps18 PE=2 SV=1	62	Tag2	18	12476	62
117	Ribosomal proteins	D3Z6C3	Rps3a2	40S ribosomal protein S3a OS=Mus musculus GN=Rps3a2 PE=3 SV=1	54	Tag3	19	29806	54
118	Ribosomal proteins	P62754	Rps6	40S ribosomal protein S6 OS=Mus musculus GN=Rps6 PE=1 SV=1	67	Tag3	14	28663	67
119	RNA/DNA binding	Q9JKX4	Aatf	Protein AATF OS=Mus musculus GN=Aatf PE=1 SV=1	86	Tag6	14	59446	86
120	RNA/DNA binding	Q6P542	Abcf1	ATP-binding cassette sub-family F member 1 OS=Mus musculus GN=Abcf1 PE=1 SV=1	102	Tag9	19	94887	102
121	RNA/DNA binding	D3YYE1	Anp32a	Acidic leucine-rich nuclear phosphoprotein 32 family member A (Fragment) OS=Mus musculus GN=	35	Tag5	130	22940	35
122	RNA/DNA binding	P63154	Crnkl1	Crooked neck-like protein 1 OS=Mus musculus GN=Crnkl1 PE=2 SV=1	51	Tag6	20	83363	51
123	RNA/DNA binding	Q80Y44	Ddx10	Probable ATP-dependent RNA helicase DDX10 OS=Mus musculus GN=Ddx10 PE=1 SV=2	31	Tag9	44	100676	31
124	RNA/DNA binding	F8WJA0	Ddx24	ATP-dependent RNA helicase DDX24 OS=Mus musculus GN=Ddx24 PE=2 SV=1	554	Tag9	3	101312	554
125	RNA/DNA binding	Q9D0R4	Ddx56	Probable ATP-dependent RNA helicase DDX56 OS=Mus musculus GN=Ddx56 PE=2 SV=1	38	Tag4	87	61174	38
126	RNA/DNA binding	Q8R2M2	Dnttip2	Deoxynucleotidyltransferase terminal-interacting protein 2 OS=Mus musculus GN=Dnttip2 PE=1 SV=	55	Tag9	30	84226	55
127	RNA/DNA binding	P84089	Erh	Enhancer of rudimentary homolog OS=Mus musculus GN=Erh PE=1 SV=1	34	Tag1	51	12251	34
128	RNA/DNA binding	Q8BK35	Gltscr2	MCG2065, isoform CRA_c OS=Mus musculus GN=Gltscr2 PE=2 SV=1	33	Tag4	100	55759	33
129	RNA/DNA binding	D3YYT1	Glyr1	Putative oxidoreductase GLYR1 OS=Mus musculus GN=Glyr1 PE=4 SV=1	77	Tag4	46	60397	77
130	RNA/DNA binding	Q4VBD9	Gzf1	GDNF-inducible zinc finger protein 1 OS=Mus musculus GN=Gzf1 PE=1 SV=2	32	Tag11	92	79482	32
131	RNA/DNA binding	P38647	Hspa9	Stress-70 protein, mitochondrial OS=Mus musculus GN=Hspa9 PE=1 SV=3	117	Tag6	10	73416	117
132	RNA/DNA binding	Q3UFM5	Nom1	Nucleolar MIF4G domain-containing protein 1 OS=Mus musculus GN=Nom1 PE=2 SV=2	73	Tag8	32	95900	73
133	RNA/DNA binding	A2A813	Park7	Protein DJ-1 OS=Mus musculus GN=Park7 PE=2 SV=1	35	Tag5	129	18462	35
134	RNA/DNA binding	Q3UHX2	Pdap1	28 kDa heat- and acid-stable phosphoprotein OS=Mus musculus GN=Pdap1 PE=1 SV=1	32	Tag5	139	20593	32
135	RNA/DNA binding	B1AXW4	Prdx1	Peroxiredoxin-1 (Fragment) OS=Mus musculus GN=Prdx1 PE=2 SV=1	31	Tag5	144	13623	31
136	RNA/DNA binding	Q8BG13	Rbm3	Putative RNA-binding protein 3 OS=Mus musculus GN=Rbm3 PE=2 SV=1	36	Tag8	59	16751	36
137	RNA/DNA binding	F7AA45	Rbm39	RNA-binding protein 39 (Fragment) OS=Mus musculus GN=Rbm39 PE=4 SV=1	40	Tag5	115	33918	40
138	RNA/DNA binding	A2A7F3	Rlf	Protein Rlf (Fragment) OS=Mus musculus GN=Rlf PE=2 SV=1	53	Tag12	34	12482	53
139	RNA/DNA binding	Q80TE0	Rpap1	RNA polymerase II-associated protein 1 OS=Mus musculus GN=Rpap1 PE=1 SV=2	33	Tag1	53	155172	33
140	RNA/DNA binding	Q9Z315	Sart1	U4/U6.U5 tri-snRNP-associated protein 1 OS=Mus musculus GN=Sart1 PE=2 SV=1	213	Tag9	11	90830	213
141	RNA/DNA binding	Q9CY58	Serbp1	Plasminogen activator inhibitor 1 RNA-binding protein OS=Mus musculus GN=Serbp1 PE=1 SV=2	37	Tag11	83	44687	37
142	RNA/DNA binding	Q8R2K3	Ssbp1	Single-stranded DNA-binding protein OS=Mus musculus GN=Ssbp1 PE=2 SV=1	129	Tag1	17	17146	129
143	RNA/DNA binding	P47226-2	Tes	Isoform TES1 of Testin OS=Mus musculus GN=Tes	32	Tag1	55	41744	32
144	RNA/DNA binding	Q0P678	Zc3h18	Zinc finger CCCH domain-containing protein 18 OS=Mus musculus GN=Zc3h18 PE=1 SV=1	34	Tag10	45	105631	34
145	RNA/DNA binding	F6QZB1	Zcchc10	Zinc finger CCHC domain-containing protein 10 (Fragment) OS=Mus musculus GN=Zcchc10 PE=4	41	Tag3	24	3434	41
146	RNA/DNA binding	A2AQR3	Zfp64	Zinc finger protein 64 OS=Mus musculus GN=Zfp64 PE=2 SV=1	56	Tag11	69	71668	56
147	RNA/DNA binding	Q99LI5	Znf281	Zinc finger protein 281 OS=Mus musculus GN=Znf281 PE=1 SV=1	200	Tag9	14	96625	200
148	RNA/DNA binding	Q99LH4	Znf672	Zinc finger protein 672 OS=Mus musculus GN=Znf672 PE=2 SV=1	31	Tag9	46	52121	31
149	rRNA genesis	Q8CI11	Gnl3	Guanine nucleotide-binding protein-like 3 OS=Mus musculus GN=Gnl3 PE=1 SV=2	123	Tag5	40	60749	123
150	rRNA genesis	Q99ME9	Gtpbp4	Nucleolar GTP-binding protein 1 OS=Mus musculus GN=Gtpbp4 PE=2 SV=3	32	Tag6	31	74066	32
151	rRNA genesis	A2ANY6	Mdn1	Midasin OS=Mus musculus GN=Mdn1 PE=2 SV=1	61	Tag14	11	629945	61
152	rRNA genesis	Q9D6Z1	Nop56	Nucleolar protein 56 OS=Mus musculus GN=Nop56 PE=1 SV=2	157	Tag5	33	64424	157
153	rRNA genesis	Q9DBY8	Nvl	Nuclear valosin-containing protein-like OS=Mus musculus GN=Nvl PE=1 SV=1	516	Tag8	9	94417	516
154	Metabolism	B8JK56	Adcy3	Adenylate cyclase type 3 OS=Mus musculus GN=Adcy3 PE=3 SV=1	53	Tag11	71	128914	53
155	Metabolism	Q5SSM3	Arhgap44	Rho GTPase-activating protein 44 OS=Mus musculus GN=Arhgap44 PE=2 SV=1	30	Tag2	32	88937	30
156	Metabolism	D3YX85	Asap2	Arf-GAP with SH3 domain, ANK repeat and PH domain-containing protein 2 OS=Mus musculus GN=	44	Tag7	85	106339	44
157	Metabolism	P15327	Bpgm	Bisphosphoglycerate mutase OS=Mus musculus GN=Bpgm PE=2 SV=2	44	Tag5	104	29960	44
158	Metabolism	P16858	Gapdh	Glyceraldehyde-3-phosphate dehydrogenase OS=Mus musculus GN=Gapdh PE=1 SV=2	120	Tag11	46	35787	120
159	Metabolism	Q8CFB4	Gbp5	Guanylate-binding protein 5 OS=Mus musculus GN=Gbp5 PE=1 SV=2	30	Tag2	35	66888	30
160	Metabolism	B7FAU8	Gdi1	Guanosine diphosphate (GDP) dissociation inhibitor 1 (Fragment) OS=Mus musculus GN=Gdi1 PE=	48	Tag5	95	16852	48
161	Metabolism	P50153	Gng4	Guanine nucleotide-binding protein G(I)/G(S)/G(O) subunit gamma-4 OS=Mus musculus GN=Gng4	32	Tag1	58	8399	32
162	Metabolism	F7ALS6	Got1	Aspartate aminotransferase, cytoplasmic (Fragment) OS=Mus musculus GN=Got1 PE=4 SV=1	42	Tag5	109	20365	42
163	Metabolism	G3UWY0	Guf1	Translation factor Guf1, mitochondrial (Fragment) OS=Mus musculus GN=Guf1 PE=2 SV=1	35	Tag11	89	63137	35
164	Metabolism	Q8BFZ6	Idi2	Isopentenyl-diphosphate Delta-isomerase 2 OS=Mus musculus GN=Idi2 PE=2 SV=1	31	Tag1	60	26647	31
165	Metabolism	Q9Z2L6	Minpp1	Multiple inositol polyphosphate phosphatase 1 OS=Mus musculus GN=Minpp1 PE=1 SV=3	33	Tag12	47	54503	33
166	Metabolism	Q3UW66	Mpst	Sulfurtransferase OS=Mus musculus GN=Mpst PE=2 SV=1	30	Tag5	152	33077	30
167	Metabolism	A2AUP1	Pfkfb3	Protein Pfkfb3 OS=Mus musculus GN=Pfkfb3 PE=2 SV=1	32	Tag11	95	59633	32
168	Metabolism	Q8C605	Pfkp	6-phosphofructokinase OS=Mus musculus GN=Pfkp PE=2 SV=1	82	Tag7	50	85492	82
169	Metabolism	O08709	Prdx6	Peroxiredoxin-6 OS=Mus musculus GN=Prdx6 PE=1 SV=3	74	Tag7	57	24855	74
170	Metabolism	F6SLR4	Spock2	Testican-2 OS=Mus musculus GN=Spock2 PE=4 SV=1	30	Tag1	63	46829	30
171	Metabolism	Q93092	Taldo1	Transaldolase OS=Mus musculus GN=Taldo1 PE=1 SV=2	37	Tag7	90	37363	37
172	Metabolism	Q9CQV8	Ywhab	14-3-3 protein beta/alpha OS=Mus musculus GN=Ywhab PE=1 SV=3	147	Tag11	35	28069	147
173	Metabolism	P13634	Ca1	Carbonic anhydrase 1 OS=Mus musculus GN=Ca1 PE=2 SV=4	65	Tag7	65	28313	65
174	Miscellaneous	P14211	Calr	Calreticulin OS=Mus musculus GN=Calr PE=1 SV=1	33	Tag7	92	47965	33
175	Miscellaneous	F8VQ93	Phip	PH-interacting protein OS=Mus musculus GN=Phip PE=4 SV=1	754	Tag12	4	206595	754
176	Miscellaneous	Q60692	Psmb6	Proteasome subunit beta type-6 OS=Mus musculus GN=Psmb6 PE=1 SV=3	30	Tag13	58	25362	30
177	Miscellaneous	Q9WVM1	Racgap1	Rac GTPase-activating protein 1 OS=Mus musculus GN=Racgap1 PE=2 SV=1	37	Tag6	27	70114	37
178	Miscellaneous	Q14AA6	1700009N1	MCG49183 OS=Mus musculus GN=1700009N14Rik PE=2 SV=1	32	Tag7	93	24342	32
179	Miscellaneous	D3Z1D3	3425401B1	Protein 3425401B19Rik OS=Mus musculus GN=3425401B19Rik PE=4 SV=1	30	Tag9	49	154383	30
180	Miscellaneous	E9PVG8	9530053A0	Protein 9530053A07Rik OS=Mus musculus GN=9530053A07Rik PE=4 SV=1	64	Tag4	57	280044	64
181	Miscellaneous	P29755	Agtr1b	Type-1B angiotensin II receptor OS=Mus musculus GN=Agtr1b PE=2 SV=1	30	Tag9	47	40923	30
182	Miscellaneous	P29699	Ahsg	Alpha-2-HS-glycoprotein OS=Mus musculus GN=Ahsg PE=1 SV=1	166	Tag11	32	37302	166
183	Miscellaneous	Q925I1	Atad3	ATPase family AAA domain-containing protein 3 OS=Mus musculus GN=Atad3 PE=1 SV=1	100	Tag5	51	66701	100
184	Miscellaneous	Q8R2U2	BC027231	Protein BC027231 OS=Mus musculus GN=BC027231 PE=2 SV=1	30	Tag2	34	63354	30
185	Miscellaneous	D3YYQ4	Car1	Protein Car1 (Fragment) OS=Mus musculus GN=Car1 PE=2 SV=1	45	Tag4	72	9866	45
186	Miscellaneous	Q6TDU8	Casc1	Cancer susceptibility candidate protein 1 OS=Mus musculus GN=Casc1 PE=2 SV=1	59	Tag11	66	84912	59
187	Miscellaneous	O08707	Ccbp2	Chemokine-binding protein 2 OS=Mus musculus GN=Ccbp2 PE=2 SV=2	40	Tag3	25	43178	40
188	Miscellaneous	Q5M8N0	Cnrip1	CB1 cannabinoid receptor-interacting protein 1 OS=Mus musculus GN=Cnrip1 PE=1 SV=1	38	Tag5	117	18601	38
189	Miscellaneous	G3UXG1	Cp	Ceruloplasmin (Fragment) OS=Mus musculus GN=Cp PE=2 SV=1	30	Tag5	153	13556	30
190	Miscellaneous	D3Z7F7	Cpd1	Calcineurin-like phosphoesterase domain-containing protein 1 (Fragment) OS=Mus musculus GN=(	36	Tag5	127	27198	36
191	Miscellaneous	Q8BG79	Cwf19l2	CWF19-like protein 2 OS=Mus musculus GN=Cwf19l2 PE=2 SV=1	43	Tag9	41	103108	43
192	Miscellaneous	P16381	D1Pas1	Putative ATP-dependent RNA helicase Pl10 OS=Mus musculus GN=D1Pas1 PE=1 SV=1	38	Tag6	25	73095	38
193	Miscellaneous	A2AQ53	Fbn1	Fibrillin-1 OS=Mus musculus GN=Fbn1 PE=4 SV=1	37	Tag2	26	312083	37
194	Miscellaneous	Q9Z1W4	Gdf11	Growth/differentiation factor 11 OS=Mus musculus GN=Gdf11 PE=2 SV=1	32	Tag11	93	44918	32
195	Miscellaneous	H3BLB0	Gm13150	Protein Gm13150 OS=Mus musculus GN=Gm13150 PE=4 SV=1	30	Tag7	101	77412	30
196	Miscellaneous	G3UYJ7	Gm20441	Protein Gm20441 (Fragment) OS=Mus musculus GN=Gm20441 PE=4 SV=1	58	Tag7	71	28616	58
197	Miscellaneous	F6RGI3	Gm340	Protein Gm340 OS=Mus musculus GN=Gm340 PE=4 SV=1	50	Tag12	35	136094	50
198	Miscellaneous	A2AE89	Gstm1	Glutathione S-transferase Mu 1 (Fragment) OS=Mus musculus GN=Gstm1 PE=2 SV=1	30	Tag11	100	23521	30
199	Miscellaneous	P62748	Hpcal1	Hippocalcin-like protein 1 OS=Mus musculus GN=Hpcal1 PE=2 SV=2	31	Tag10	55	22324	31
200	Miscellaneous	Q3U6C5	Kcnab1	Voltage-gated potassium channel subunit beta-1 OS=Mus musculus GN=Kcnab1 PE=2 SV=1	35	Tag14	17	33177	35
201	Miscellaneous	Q61074	Ppm1g	Protein phosphatase 1G OS=Mus musculus GN=Ppm1g PE=2 SV=3	52	Tag5	91	58691	52
202	Miscellaneous	Q8BP92	Rcn2	Reticulocalbin-2 OS=Mus musculus GN=Rcn2 PE=2 SV=1	90	Tag4	37	37248	90
203	Miscellaneous	B1AYD3	Syndig1	Synapse differentiation-inducing gene protein 1 (Fragment) OS=Mus musculus GN=Syndig1 PE=2 S	31	Tag11	96	7859	31
204	Miscellaneous	Q6ZPJ0	Tex2	Testis-expressed sequence 2 protein OS=Mus musculus GN=Tex2 PE=1 SV=2	36	Tag1	49	125147	36
205	Miscellaneous	E9Q759	Vmn2r14	Protein Vmn2r14 OS=Mus musculus GN=Vmn2r14 PE=3 SV=1	32	Tag1	57	96121	32
206	Miscellaneous	Q9Z2U2	Zfp292	Zinc finger protein 292 OS=Mus musculus GN=Zfp292 PE=2 SV=2	32	Tag13	54	300858	32
207	Miscellaneous	Q9CZP3	Zfp655	Protein Zfp655 OS=Mus musculus GN=Zfp655 PE=2 SV=1	58	Tag5	85	63333	58

Table S1B

Software:		Mascot version 2.4.0		
Database		Uniprot_mouse (50,807 sequences; 24,405,143 residues)		
Enzyme		Trypsin		
Maximum Missed Cleav		3		
Mass values		Monoisotopic		
Protein score cuttoff		30		
Peptide Ions score cut-o		20		
Instrument type		ESI-TRAP (Orbitrap Elite)		

Sample ID		46C-tag		
prot_hit_num	prot_acc	prot_desc	prot_score	prot_MW
1	P62806	Histone H4 OS=Mus musculus GN=Hist1h4a PE=1 SV=2	697	11360
2	Q8BFU2	Histone H2A type 3 OS=Mus musculus GN=Hist3h2a PE=1 SV=3	570	14113
3	Q6GSS7	Histone H2A type 2-A OS=Mus musculus GN=Hist2h2aa1 PE=1 SV=3	498	14087
4	P27661	Histone H2A.x OS=Mus musculus GN=H2afx PE=1 SV=2	411	15133
5	Q6ZWY9	Histone H2B type 1-C/E/G OS=Mus musculus GN=Hist1h2bc PE=1 SV=3	292	13898
6	P68433	Histone H3.1 OS=Mus musculus GN=Hist1h3a PE=1 SV=2	186	15394
7	E0CZ27	Histone H3 (Fragment) OS=Mus musculus GN=H3f3a PE=2 SV=1	176	13314
8	P0C0S6	Histone H2A.Z OS=Mus musculus GN=H2afz PE=1 SV=2	162	13545
9	P56480	ATP synthase subunit beta, mitochondrial OS=Mus musculus GN=Atp5b PE=1 SV=2	129	56265
10	E9Q0F0	Protein Krt78 OS=Mus musculus GN=Krt78 PE=2 SV=1	100	112194
11	P07724	Serum albumin OS=Mus musculus GN=Alb PE=1 SV=3	97	68648
12	P17897	Lysozyme C-1 OS=Mus musculus GN=Lyz1 PE=1 SV=1	92	16783
13	Q792Z1	MCG140784 OS=Mus musculus GN=Try10 PE=2 SV=1	90	26204
14	Q9CPN9	Protein 2210010C04Rik OS=Mus musculus GN=2210010C04Rik PE=2 SV=1	77	26405
15	G3UYI5	Protein mago nashi homolog 2 (Fragment) OS=Mus musculus GN=Magohb PE=2 SV=1	68	13376
16	G3X963	ATPase family AAA domain-containing protein 2 OS=Mus musculus GN=Atad2 PE=4 SV=1	63	155188
17	P62264	40S ribosomal protein S14 OS=Mus musculus GN=Rps14 PE=2 SV=3	56	16263
18	Q9Z1R9	MCG124046 OS=Mus musculus GN=Prss1 PE=2 SV=1	52	26118
19	Q504P4	Heat shock cognate 71 kDa protein OS=Mus musculus GN=Hspa8 PE=2 SV=1	50	68736
20	A2ANY8	Ankyrin repeat domain-containing protein 6 OS=Mus musculus GN=Ankrd6 PE=2 SV=1	38	71154
21	P62878	E3 ubiquitin-protein ligase RBX1 OS=Mus musculus GN=Rbx1 PE=1 SV=1	37	12266
22	E9PWG9	Mitogen-activated protein kinase kinase kinase 5 OS=Mus musculus GN=Map3k5 PE=2 SV=1	32	153485
23	Q3UH13	Tubby-related protein 4 OS=Mus musculus GN=Tulp4 PE=2 SV=1	31	147808
24	E0CXE3	Contactin-6 OS=Mus musculus GN=Cntn6 PE=2 SV=1	30	105450

Sample ID		46C		
prot_hit_num	prot_acc	prot_desc	prot_score	prot_MW
1	P17897	Lysozyme C-1 OS=Mus musculus GN=Lyz1 PE=1 SV=1	88	16783
2	P0C0S6	Histone H2A.Z OS=Mus musculus GN=H2afz PE=1 SV=2	75	13545
3	E9Q0F0	Protein Krt78 OS=Mus musculus GN=Krt78 PE=2 SV=1	74	112194
4	P07724	Serum albumin OS=Mus musculus GN=Alb PE=1 SV=3	72	68648
5	Q8CBB6	Histone H2B OS=Mus musculus GN=Gm13646 PE=2 SV=1	55	14879
6	Q792Z1	MCG140784 OS=Mus musculus GN=Try10 PE=2 SV=1	47	26204
7	Q9CPN9	Protein 2210010C04Rik OS=Mus musculus GN=2210010C04Rik PE=2 SV=1	45	26405
8	Q9Z1R9	MCG124046 OS=Mus musculus GN=Prss1 PE=2 SV=1	45	26118
9	D3YX85	Arf-GAP with SH3 domain, ANK repeat and PH domain-containing protein 2 OS=Mus musculus GN=Asap2 PE=2 S	41	106339
10	F6XWB2	Uncharacterized protein OS=Mus musculus GN=Igkv1-115 PE=4 SV=1	40	12420
11	F6QFD1	cAMP-specific 3~,5~-cyclic phosphodiesterase 4D (Fragment) OS=Mus musculus GN=Pde4d PE=3 SV=1	36	85498
12	D3Z7R9	Cell cycle progression 1, isoform CRA_d OS=Mus musculus GN=Ccpg1 PE=4 SV=1	31	89078
13	Q3UH13	Tubby-related protein 4 OS=Mus musculus GN=Tulp4 PE=2 SV=1	31	147808
14	M0QWL0	Protein Myh13 (Fragment) OS=Mus musculus GN=Myh13 PE=4 SV=1	31	25760

Table S1C

	acetylated site	mod:unmod	acetylated site	mod:unmod
46C-tag	H4K5	5\2	H3K9	11\6
	H4K8	7\2	H3K14	17\2
	H4K12	9\0	H3K18	5\6
	H4K16	7\2	H3K23	8\3
			H3K79	4\6
46C	Acetylated peptides were not identified.			

Histone acetylated spectra

(peptide ion score cutoff 20 and checked manually)

**46C-tag_H3**		
10 - 18	R.KSTGGKAPR.K	Acetyl (K) (Ions score 25)
10 - 18	R.KSTGGKAPR.K	Acetyl (K); Methyl (K) (Ions score 38)
10 - 18	R.KSTGGKAPR.K	Acetyl (K); Methyl (K) (Ions score 38)
10 - 18	R.KSTGGKAPR.K	Acetyl (K); Dimethyl (K) (Ions score 23)
10 - 18	R.KSTGGKAPR.K	Acetyl (K); Dimethyl (K) (Ions score 57)
10 - 18	R.KSTGGKAPR.K	Acetyl (K); Dimethyl (K) (Ions score 42)
10 - 18	R.KSTGGKAPR.K	2 Acetyl (K) (Ions score 26)
10 - 18	R.KSTGGKAPR.K	2 Acetyl (K) (Ions score 30)
10 - 18	R.KSTGGKAPR.K	2 Acetyl (K) (Ions score 25)
10 - 18	R.KSTGGKAPR.K	2 Acetyl (K) (Ions score 29)
10 - 18	R.KSTGGKAPR.K	2 Acetyl (K) (Ions score 33)
10 - 18	R.KSTGGKAPR.K	2 Acetyl (K) (Ions score 41)
10 - 18	R.KSTGGKAPR.K	2 Acetyl (K) (Ions score 23)
10 - 18	R.KSTGGKAPR.K	2 Acetyl (K) (Ions score 27)
10 - 18	R.KSTGGKAPR.K	2 Acetyl (K) (Ions score 24)
10 - 18	R.KSTGGKAPR.K	Acetyl (K); Trimethyl (K) (Ions score 33)
10 - 18	R.KSTGGKAPR.K	Acetyl (K); Trimethyl (K) (Ions score 32)
11 - 18	K.STGGKAPR.K	Acetyl (K) (Ions score 33)
11 - 18	K.STGGKAPR.K	Acetyl (K) (Ions score 28)
19 - 24	R.KQLATK.A	Acetyl (K) (Ions score 24)
19 - 24	R.KQLATK.A	Acetyl (K) (Ions score 40)
19 - 24	R.KQLATK.A	Acetyl (K) (Ions score 36)
19 - 27	R.KQLATKAAR.K	Acetyl (K) (Ions score 25)
19 - 27	R.KQLATKAAR.K	Acetyl (K) (Ions score 28)
19 - 27	R.KQLATKAAR.K	Acetyl (K) (Ions score 39)
19 - 27	R.KQLATKAAR.K	Acetyl (K) (Ions score 42)
19 - 27	R.KQLATKAAR.K	2 Acetyl (K) (Ions score 50)
19 - 27	R.KQLATKAAR.K	2 Acetyl (K) (Ions score 49)
20 - 27	K.QLATKAAR.K	Acetyl (K) (Ions score 32)
20 - 27	K.QLATKAAR.K	Acetyl (K) (Ions score 22)
74 - 84	R.EIAQDFKTDLR.F	Acetyl (K) (Ions score 43)
74 - 84	R.EIAQDFKTDLR.F	Acetyl (K) (Ions score 43)
74 - 84	R.EIAQDFKTDLR.F	Acetyl (K) (Ions score 48)
74 - 84	R.EIAQDFKTDLR.F	Acetyl (K) (Ions score 56)
74 - 80	R.EIAQDFK.T	(Ions score 24)
74 - 80	R.EIAQDFK.T	(Ions score 28)
74 - 84	R.EIAQDFKTDLR.F	(Ions score 32)
74 - 84	R.EIAQDFKTDLR.F	(Ions score 44)
74 - 84	R.EIAQDFKTDLR.F	(Ions score 27)
74 - 84	R.EIAQDFKTDLR.F	(Ions score 47)
**46C-Tag_H4**		
5 - 17	R.GKGGKGLGKGGAK.R	3 Acetyl (K) (Ions score 57)
5 - 17	R.GKGGKGLGKGGAK.R	3 Acetyl (K) (Ions score 49)
5 - 18	R.GKGGKGLGKGGAKR.H	4 Acetyl (K) (Ions score 72)
5 - 18	R.GKGGKGLGKGGAKR.H	4 Acetyl (K) (Ions score 30)
5 - 18	R.GKGGKGLGKGGAKR.H	4 Acetyl (K) (Ions score 47)
7 - 18	K.GGKGLGKGGAKR.H	3 Acetyl (K) (Ions score 49)
7 - 18	K.GGKGLGKGGAKR.H	3 Acetyl (K) (Ions score 53)
10 - 18	K.GLGKGGAKR.H	2 Acetyl (K) (Ions score 43)
10 - 18	K.GLGKGGAKR.H	2 Acetyl (K) (Ions score 40)

Table S2 (related to Figure 3C). Analysis of the proteins co-purified with chromatin-bound Atad2 (whole identification approach). List of proteins identified in a single gel slice after short migration of $46C^{tag}$ but not in 46C extracts after tandem affinity purification was established and proteins identified in both ChIP-proteomics approaches are indicated and classified in functional categories.

CPDB_groups	prot_acc	GeneSymbol	prot_desc	Score (tag or tag-46C)	Band	hit_num	prot_MW	RatioScoreTag/46C
**Atad2**	**G3X963**	**Atad2**	**ATPase family AAA domain-containing protein 2 OS=Mus musculus GN=Atad2**	**16130**	**Tag11**	**1**	**155188**	**171**
	**E9Q166**	**Atad2b**	**Protein Atad2b OS=Mus musculus GN=Atad2b PE=2 SV=1**	**12132**	**Tag11**	**2**	**164621**	**90**
Chromatin binding/remodelling and complexes	Q6PGG6	Gnl3l	Guanine nucleotide-binding protein-like 3-like protein OS=Mus musculus GN=Gnl3l PE	170	Tag5	31	65153	170
	Q7TPV4	Mybbp1a	Myb-binding protein 1A OS=Mus musculus GN=Mybbp1a PE=1 SV=2	191	Tag10	14	151942	191
	Q91ZW3	Smarca5	SWI/SNF-related matrix-associated actin-dependent regulator of chromatin subfamily A	147	Tag10	17	121550	147
	Q62318	Trim28	Transcription intermediary factor 1-beta OS=Mus musculus GN=Trim28 PE=1 SV=3	330	Tag8	11	88791	330
Cohesins	Q7TSY8	Sgol2	Shugoshin-like 2 OS=Mus musculus GN=Sgol2 PE=1 SV=1	643	Tag10	4	130196	643
Histone acetylation	D3YYI8	Gm10093	Histone deacetylase OS=Mus musculus GN=Gm10093 PE=3 SV=1	36	Tag4	95	55013	36
Histone methylation	D3Z774	Ezh2	Histone-lysine N-methyltransferase EZH2 OS=Mus musculus GN=Ezh2 PE=2 SV=1	63	Tag7	66	80945	63
Histones	P0C0S6	H2afz	Histone H2A.Z OS=Mus musculus GN=H2afz PE=1 SV=2	361	Tag2	9	13545	7
	P43276	Hist1h1b	Histone H1.5 OS=Mus musculus GN=Hist1h1b PE=1 SV=2	46	Tag7	82	22562	46
	P43277	Hist1h1d	Histone H1.3 OS=Mus musculus GN=Hist1h1d PE=1 SV=2	113	Tag5	44	22086	113
	P43274	Hist1h1e	Histone H1.4 OS=Mus musculus GN=Hist1h1e PE=1 SV=2	107	Tag5	48	21964	107
	Q6ZWY9	Hist1h2bc	Histone H2B type 1-C/E/G OS=Mus musculus GN=Hist1h2bc PE=1 SV=3	1667	Tag2	2	13898	17
	P62806	Hist1h4a	Histone H4 OS=Mus musculus GN=Hist1h4a PE=1 SV=2	2067	Tag1	1	11360	4
	Q6GSS7	Hist2h2aa1	Histone H2A type 2-A OS=Mus musculus GN=Hist2h2aa1 PE=1 SV=3	701	Tag2	7	14087	12
	Q64522	Hist2h2ab	Histone H2A type 2-B OS=Mus musculus GN=Hist2h2ab PE=1 SV=3	99	Tag1	22	14005	99
Kinesins	Q80WE4	Kif20b	Kinesin-like protein KIF20B OS=Mus musculus GN=Kif20b PE=1 SV=3	260	Tag12	12	203389	260
	E9Q5G3	Kif23	Protein Kif23 OS=Mus musculus GN=Kif23 PE=2 SV=1	53	Tag9	32	108707	53
	Q922S8	Kif2c	Kinesin-like protein KIF2C OS=Mus musculus GN=Kif2c PE=1 SV=1	77	Tag7	55	81034	77
	P33174	Kif4	Chromosome-associated kinesin KIF4 OS=Mus musculus GN=Kif4 PE=1 SV=3	507	Tag10	6	139432	507
NuclearStructure	Q8K224	Nat10	N-acetyltransferase 10 OS=Mus musculus GN=Nat10 PE=2 SV=1	212	Tag9	12	115346	212
	E9Q7G0	Numa1	Protein Numa1 OS=Mus musculus GN=Numa1 PE=2 SV=1	2272	Tag12	2	235487	2272
	Q9CXS4	Cenpv	Centromere protein V OS=Mus musculus GN=Cenpv PE=2 SV=2	173	Tag3	6	27524	173
Sphase & replication	E9PVX6	Mki67	Protein Mki67 OS=Mus musculus GN=Mki67 PE=2 SV=1	372	Tag13	7	350650	372
	Q8BUI3	LRWD1	Leucine-rich repeat and WD repeat-containing protein 1 OS=Mus musculus GN=LRWD	444	Tag5	11	71549	444
	Q9Z1N2	Orc1	Origin recognition complex subunit 1 OS=Mus musculus GN=Orc1 PE=1 SV=2	414	Tag8	10	95043	414
	Q9JK30	Orc3	Origin recognition complex subunit 3 OS=Mus musculus GN=Orc3 PE=1 SV=1	139	Tag6	7	82289	139
	G3UWX1	Rfc1	Replication factor C subunit 1 OS=Mus musculus GN=Rfc1 PE=2 SV=1	457	Tag10	8	125787	457
	Q6PR54	Rif1	Telomere-associated protein RIF1 OS=Mus musculus GN=Rif1 PE=1 SV=2	744	Tag13	5	266063	744
Repair	Q3U1J4	Ddb1	DNA damage-binding protein 1 OS=Mus musculus GN=Ddb1 PE=1 SV=2	66	Tag9	26	126772	66
	P43247	Msh2	DNA mismatch repair protein Msh2 OS=Mus musculus GN=Msh2 PE=2 SV=1	544	Tag9	4	104085	544
	P54276	Msh6	DNA mismatch repair protein Msh6 OS=Mus musculus GN=Msh6 PE=1 SV=3	318	Tag11	16	150989	318
	Q921K2	Parp1	Poly (ADP-ribose) polymerase family, member 1 OS=Mus musculus GN=Parp1 PE=2	40	Tag9	42	112650	40
Splicing	A2AH85	Eftud2	116 kDa U5 small nuclear ribonucleoprotein component OS=Mus musculus GN=Eftud2	31	Tag9	45	109422	31
	G3XA10	Hnrnpu	Heterogeneous nuclear ribonucleoprotein U OS=Mus musculus GN=Hnrnpu PE=4 SV	305	Tag9	7	86751	305
	Q91YR7	Prpf6	Pre-mRNA-processing factor 6 OS=Mus musculus GN=Prpf6 PE=2 SV=1	478	Tag9	5	106655	478
Ribosomal proteins	P47963	Rpl13	60S ribosomal protein L13 OS=Mus musculus GN=Rpl13 PE=2 SV=3	118	Tag3	9	24290	118
	P62918	Rpl8	60S ribosomal protein L8 OS=Mus musculus GN=Rpl8 PE=2 SV=2	55	Tag3	18	28007	55
	G3UZW2	Rps18	40S ribosomal protein S18 OS=Mus musculus GN=Rps18 PE=2 SV=1	62	Tag2	18	12476	62
RNA/DNA binding	P84089	Erh	Enhancer of rudimentary homolog OS=Mus musculus GN=Erh PE=1 SV=1	34	Tag1	51	12251	34
	D3YYT1	Glyr1	Putative oxidoreductase GLYR1 OS=Mus musculus GN=Glyr1 PE=4 SV=1	77	Tag4	46	60397	77
	Q3UFM5	Nom1	Nucleolar MIF4G domain-containing protein 1 OS=Mus musculus GN=Nom1 PE=2 SV	73	Tag8	32	95900	73
	Q9Z315	Sart1	U4/U6.U5 tri-snRNP-associated protein 1 OS=Mus musculus GN=Sart1 PE=2 SV=1	213	Tag9	11	90830	213
	Q0P678	Zc3h18	Zinc finger CCCH domain-containing protein 18 OS=Mus musculus GN=Zc3h18 PE=1	34	Tag10	45	105631	34
	F6QZB1	Zcchc10	Zinc finger CCHC domain-containing protein 10 (Fragment) OS=Mus musculus GN=Z	41	Tag3	24	3434	41
rRNA genesis	Q8CI11	Gnl3	Guanine nucleotide-binding protein-like 3 OS=Mus musculus GN=Gnl3 PE=1 SV=2	123	Tag5	40	60749	123
	Q99ME9	Gtpbp4	Nucleolar GTP-binding protein 1 OS=Mus musculus GN=Gtpbp4 PE=2 SV=3	32	Tag6	31	74066	32
	Q9D6Z1	Nop56	Nucleolar protein 56 OS=Mus musculus GN=Nop56 PE=1 SV=2	157	Tag5	33	64424	157
	Q9DBY8	Nvl	Nuclear valosin-containing protein-like OS=Mus musculus GN=Nvl PE=1 SV=1	516	Tag8	9	94417	516
Miscellaneous	F8VQ93	Phip	PH-interacting protein OS=Mus musculus GN=Phip PE=4 SV=1	754	Tag12	4	206595	754

Table S3 (related to Figure 6C). Differentiation phenotype in shAtad2 Embryoid bodies (EBs). Values in μm used for box plots shown in Figure 6C.

	diameter (micro meter)	
No	Control	ATAD2 KD
1	859.469	711.076
3	925.813	633.687
4	927.418	724.706
5	896.791	673.827
6	869.753	716.213
7	889.79	755.766
8	923.142	678.006
9	894.562	724.678
10	983.944	671.059
11	931.853	677.516
12	877.583	702.366
13	889.79	731.137
14	911.493	714.274
15	879.25	660.814
16	873.346	697.768
17	831.552	738.148
18	923.494	675.828
19	894.902	728.052
20	853.65	681.669
21	872.465	733.958
22	905.093	789.831
23	902.816	691.905
24	911.616	757.026
25	885.438	655.881
26	909.38	760.901
27	891.903	685.713
28	870.04	677.497
29	872.837	783.312
30	918.45	668.687
31	893.474	672.211
32	929.741	670.355
33	948.575	828.825
34	883.777	682.719
35	870.535	652.324
36	864.94	654.863
37	884.831	697.972
38	879.569	657.269
39	925.602	638.161
40	925.51	686.757
41	918.484	658.31
42	887.819	690.531
43	922.526	793.095
44	939.696	670.688
45	867.634	708.758
46	851.356	687.818
47	865.633	638.263
48	875.095	624.143
	895.294	701.512
	**average**	
	**895.99425**	**698.2473958**
